# HIV-1 persistence following extremely early initiation of antiretroviral therapy (ART) during acute HIV-1 infection: An observational study

**DOI:** 10.1371/journal.pmed.1002417

**Published:** 2017-11-07

**Authors:** Timothy J. Henrich, Hiroyu Hatano, Oliver Bacon, Louise E. Hogan, Rachel Rutishauser, Alison Hill, Mary F. Kearney, Elizabeth M. Anderson, Susan P. Buchbinder, Stephanie E. Cohen, Mohamed Abdel-Mohsen, Christopher W. Pohlmeyer, Remi Fromentin, Rebecca Hoh, Albert Y. Liu, Joseph M. McCune, Jonathan Spindler, Kelly Metcalf-Pate, Kristen S. Hobbs, Cassandra Thanh, Erica A. Gibson, Daniel R. Kuritzkes, Robert F. Siliciano, Richard W. Price, Douglas D. Richman, Nicolas Chomont, Janet D. Siliciano, John W. Mellors, Steven A. Yukl, Joel N. Blankson, Teri Liegler, Steven G. Deeks

**Affiliations:** 1 Division of Experimental Medicine, University of California, San Francisco, California, United States of America; 2 Division of HIV, Infectious Diseases and Global Medicine, University of California, San Francisco, California, United States of America; 3 San Francisco Department of Public Health, San Francisco, California, United States of America; 4 Program for Evolutionary Dynamics, Harvard University, Cambridge, Massachusetts, United States of America; 5 HIV Dynamics and Replication Program, Center for Cancer Research, National Cancer Institute, Frederick, Maryland, United States of America; 6 The Wistar Institute, Philadelphia, Pennsylvania, United States of America; 7 Center for AIDS Research, Department of Medicine, Johns Hopkins University School of Medicine, Baltimore, Maryland, United States of America; 8 Centre de Recherche du CHUM and Department of Microbiology, Infectiology and Immunology, Université de Montréal, Montreal, Quebec, Canada; 9 Division of Infectious Diseases, Brigham and Women's Hospital, Boston, Massachusetts, United States of America; 10 Harvard Medical School, Boston, Massachusetts, United States of America; 11 Division of Infectious Diseases, Johns Hopkins University School of Medicine, Baltimore, Maryland, United States of America; 12 Howard Hughes Medical Institute, Baltimore, Maryland, United States of America; 13 Department of Neurology, University of California, San Francisco, California, United States of America; 14 University of California San Diego, La Jolla, California, United States of America; 15 Veterans Affairs San Diego Healthcare System, San Diego, California, United States of America; 16 Department of Medicine, University of Pittsburgh, Pittsburgh, Pennsylvania, United States of America; 17 San Francisco Veterans Affairs Medical Center, San Francisco, California, United States of America; 18 University of California, San Francisco, California, Unites States of America; Desmond Tutu HIV Centre, SOUTH AFRICA

## Abstract

**Background:**

It is unknown if extremely early initiation of antiretroviral therapy (ART) may lead to long-term ART-free HIV remission or cure. As a result, we studied 2 individuals recruited from a pre-exposure prophylaxis (PrEP) program who started prophylactic ART an estimated 10 days (Participant A; 54-year-old male) and 12 days (Participant B; 31-year-old male) after infection with peak plasma HIV RNA of 220 copies/mL and 3,343 copies/mL, respectively. Extensive testing of blood and tissue for HIV persistence was performed, and PrEP Participant A underwent analytical treatment interruption (ATI) following 32 weeks of continuous ART.

**Methods and findings:**

Colorectal and lymph node tissues, bone marrow, cerebral spinal fluid (CSF), plasma, and very large numbers of peripheral blood mononuclear cells (PBMCs) were obtained longitudinally from both participants and were studied for HIV persistence in several laboratories using molecular and culture-based detection methods, including a murine viral outgrowth assay (mVOA). Both participants initiated PrEP with tenofovir/emtricitabine during very early Fiebig stage I (detectable plasma HIV-1 RNA, antibody negative) followed by 4-drug ART intensification. Following peak viral loads, both participants experienced full suppression of HIV-1 plasma viremia. Over the following 2 years, no further HIV could be detected in blood or tissue from PrEP Participant A despite extensive sampling from ileum, rectum, lymph nodes, bone marrow, CSF, circulating CD4+ T cell subsets, and plasma. No HIV was detected from tissues obtained from PrEP Participant B, but low-level HIV RNA or DNA was intermittently detected from various CD4+ T cell subsets. Over 500 million CD4+ T cells were assayed from both participants in a humanized mouse outgrowth assay. Three of 8 mice infused with CD4+ T cells from PrEP Participant B developed viremia (50 million input cells/surviving mouse), but only 1 of 10 mice infused with CD4+ T cells from PrEP Participant A (53 million input cells/mouse) experienced very low level viremia (201 copies/mL); sequence confirmation was unsuccessful. PrEP Participant A stopped ART and remained aviremic for 7.4 months, rebounding with HIV RNA of 36 copies/mL that rose to 59,805 copies/mL 6 days later. ART was restarted promptly. Rebound plasma HIV sequences were identical to those obtained during acute infection by single-genome sequencing. Mathematical modeling predicted that the latent reservoir size was approximately 200 cells prior to ATI and that only around 1% of individuals with a similar HIV burden may achieve lifelong ART-free remission. Furthermore, we observed that lymphocytes expressing the tumor marker CD30 increased in frequency weeks to months prior to detectable HIV-1 RNA in plasma. This study was limited by the small sample size, which was a result of the rarity of individuals presenting during hyperacute infection.

**Conclusions:**

We report HIV relapse despite initiation of ART at one of the earliest stages of acute HIV infection possible. Near complete or complete loss of detectable HIV in blood and tissues did not lead to indefinite ART-free HIV remission. However, the small numbers of latently infected cells in individuals treated during hyperacute infection may be associated with prolonged ART-free remission.

## Abstract 2

Timothy Henrich and colleagues study the effect of very early antiretroviral treatment on the reservoir of HIV-infected cells in two patients.

## Author summary

**Why was this study done?:**

- Early initiation of ART following infection may limit the total body burden of HIV.
- It is not known if starting ART extremely early after HIV infection will lead to ART-free remission or cure.
- We studied 2 individuals who started ART an estimated 10 and 12 days after HIV infection with very low peak viral load measurement; extensive testing of blood and tissue for HIV persistence was performed.
- One participant stopped ART in order to test if and when HIV would rebound.

**What did the researchers find?:**

- No HIV could be definitively detected for up to 2 years in the participant who initiated ART approximately 10 days after HIV infection.
- Intermittent, very low levels of HIV were detected in blood but not tissue in the participant who initiated ART an estimated 12 days following infection.
- The participant with no detectable HIV following ART experienced viral rebound 225 days after stopping ART.

**What do these findings mean?:**

- HIV relapsed despite initiation of ART at one of the earliest stages of acute HIV infection possible.
- Near complete loss of detectable HIV in blood and tissues did not lead to indefinite ART-free HIV remission.

## Introduction

The development of a cure for HIV infection is a major public health objective [1]. Despite the ability of antiretroviral therapy (ART) to significantly reduce disease-related morbidity and mortality in HIV-1 infection, viral reservoirs persist indefinitely in latently infected cells [2]. HIV persists during ART primarily within circulating and tissue-resident, long-lived memory CD4+ T cells that harbor integrated HIV DNA; these cells are not cleared with ART and are a source of viral rebound when treatment is discontinued [3]. A major HIV eradication strategy involves aborting the initial seeding of these long-lived reservoirs by the very early initiation of ART [4,5]. For example, initiation of ART in a perinatally infected infant at 31 hours of life led to significant reductions in the viral reservoir and a significant time off ART (>2 years) before eventual viral recrudescence [6,7]. However, the impact of extremely early ART on HIV persistence and seeding the viral reservoir with the potential to prevent establishment of lifelong infection in adults is unknown.

Antiretroviral drugs initiated before HIV exposure (pre-exposure prophylaxis [PrEP]) can be an effective method of preventing HIV acquisition [8–11]. PrEP programs involve HIV antibody testing before the initiation of prophylactic ART in individuals at high risk for acquiring HIV. PrEP is typically started following negative HIV antibody or combined antibody/antigen screening in the absence of clinical symptoms [8,11]. Because there is a delay between HIV infection and when an HIV antibody or combined antibody/antigen test is reactive, PrEP may be unknowingly started in an individual who has very recently been infected with HIV. As a result, a small number of individuals may begin 2-drug ART just prior to or after the development of detectable plasma HIV-1 RNA (the transition from the "eclipse phase" to Fiebig stage I of infection) and prior to the detection of HIV antigen or antibody [12,13]. PrEP programs are therefore ideal settings in which to identify individuals treated extremely early during infection for the longitudinal study of HIV-1 reservoir persistence in blood and various tissues [4,5,14,15].

The aims of this study were to determine the impact of extremely early initiation of ART on the size of the HIV reservoir in blood and various tissues and the potential for long-term ART-free remission. As a result, we studied 2 PrEP study participants who initiated ART during emergent, unrecognized HIV infection in Fiebig stage I, with 1 individual treated just as he was transitioning out of the “eclipse phase” of HIV infection [12,13]. We describe the results of extensive tissue and blood sampling in these individuals and the result of a highly monitored treatment interruption. Using this case and our previously described recipient of an allogeneic bone marrow transplant (hematopoietic stem cell transplantation [HSCT] Participant B) [16,17], we also describe potential biomarkers for HIV reactivation during prolonged states of viremia post-ART interruption.

## Methods and materials

### Study design and population

The PrEP Demo Project was a prospective study of PrEP for men who have sex with men (MSM) in which participants were tested for HIV both by HIV antibody/antigen combination assay and by HIV RNA on the day of PrEP initiation [18]. There was no prespecified plan for the present analysis at that time. Two participants were identified in the study who had positive viral load tests performed on the day of initiation of PrEP (truvada/emtricitabine). PrEP was converted to standard ART once HIV infection was confirmed. The participants were enrolled in the longitudinal University of California San Francisco (UCSF) SCOPE study after providing written informed consent. The study was approved by the UCSF Committee on Human Research. A protocol and an analysis plan were in place prior to analytical treatment interruption (ATI). The protocol and STROBE checklist are included in the supporting information (**S1 Protocol** and **S1 STROBE Checklist**).

To more fully explore biomarkers associated with HIV reactivation after the interruption of ART, we also accessed peripheral blood mononuclear cells (PBMCs) from a previously described case of an HIV-infected adult who underwent an allogenic hematopoietic stem-cell transplantation (HSCT Participant B) [16]. All HIV reservoir studies for HSCT Participant B during ATI were conducted at commercial laboratories and cryopreserved PBMCs were not available.

### Tissue collection, apheresis, and sample processing

PBMCs and plasma were collected longitudinally either by large-volume peripheral blood draws or by leukapheresis and purified by Ficoll-Hypaque (Sigma-Aldrich) density gradient centrifugation. Colorectal and ileal tissue were collected and processed as previously described [19,20]. A whole, inguinal lymph node was obtained from each participant by surgical excision for mononuclear cell isolation and downstream HIV reservoir characterization months following combination ART initiation. Total PBMCs, purified CD4+ T cells, or CD4+ T cell subsets [naive (T_N_), central memory (T_CM_), transitional memory (T_TM_), and effector memory (T_EM_)] from blood and tissues were collected via bead-based separation (Stem Cell Technologies) or by fluorescence-activated cell sorting using previously described methods [21]. Bone marrow biopsies were performed, followed by sorting and collection of 4 cell populations: myeloid cells (CD33+Lin+/−), CD3+CD4+ T cells (CD33−Lin+CD4+), multilineage CD4+ cells (CD33−Lin−CD4dim), and Lin− cells that are not CD4+ or myeloid cells (CD33−Lin−CD4−CD34+/−). Cerebrospinal fluid (CSF) was collected by lumbar puncture and centrifuged to separate the liquid and cellular fraction for downstream HIV-1 detection, and quantification was carried out as previously described [22,23].

### HIV testing and viral reservoir characterization

HIV-1 RNA was isolated from baseline (pre-PrEP) and subsequent plasma samples followed by single-genome sequencing (SGS) of HIV-1 protease-pol (pro-pol) or a 1.5 kb portion of the envelope gene as previously described [24,25]. Population sequencing on subsequent timepoints was performed using TRUGENE (Siemens, Tarrytown NY). PBMCs and tissue-derived cells were analyzed for HIV-1 persistence using a variety of highly sensitive assays in up to 10 independent laboratories located at the UCSF, University of Montreal, University of Pittsburgh, University of California San Diego (UCSD), and Johns Hopkins University. Assays utilized were previously described and included highly sensitive quantitative PCR for total and integrated HIV-1 DNA (unspliced and total RNA) [26,27], droplet digital PCR (ddPCR) (HIV pol DNA and 2-LTR circles) [28], total virus recovery assay, quantitative viral outgrowth assay (qVOA) [29,30], Tat/Rev Induced Limiting Dilution Assay (TILDA) [31], and whole HIV genome sequencing [32]. In addition, large volume plasma was tested using the MEGA iSCA [33].

### Murine viral outgrowth assay

CD4+ T cells obtained by leukapheresis from both participants approximately 18 months following the initiation of ART were tested using the murine (humanized mouse) viral outgrowth assay (mVOA) as previously described [34]. Briefly, 530 million and 379 million CD4+ T cells were divided and injected intraperitoneally into 8–10 humanized female young adult NOD.Cg-Prkdc^scid^ Il2rg^tm1Wjl^/SzJ (NSG) mice from Jackson Labs (53 million and 50 million cells per mouse from the 2 participants). Plasma HIV-1 RNA testing was then performed following up to 5.5 weeks after engraftment on blood obtained by weekly mandibular sinus bleed not exceeding 0.5% of body weight. Mice were euthanized by CO_2_ inhalation and HIV-1 sequencing was attempted from plasma and spleen cells using cDNA and methods optimized for low HIV-1 RNA copy numbers [34]. The Johns Hopkins University Institutional Animal Care and Use Committee approved this research and it was conducted in accordance with the 8th edition of the Guide for the Care and Use of Laboratory Animals within fully AAALACi accredited animal facilities. Mice were group-housed with other mice xenografted from the same donor in microisolator caging (Allentown) and fed a commercial rodent chow (Harlan) and hyperchlorinated water ad libitum.

### ATI

A carefully monitored ATI was performed on 1 participant (PrEP Participant A) who initiated PrEP an estimated 10 days following HIV infection and had no definitive HIV detected in blood or tissues for 32 months on ART. After ATI, viral load testing was performed twice weekly using the COBAS AmpliPrep/COBAS TaqMan V.2 assay (Roche) for the first 3 months, followed by weekly testing thereafter with close clinical observation. Large-volume blood draws were also obtained monthly for the isolation of PBMCs, and plasma was obtained for further reservoir and flow cytometric testing. ART was initiated immediately after confirmation of detectable plasma HIV-1 RNA.

### Flow cytometric analysis of lymphocyte phenotypes and markers of activation, exhaustion, and proliferation

Flow cytometric testing was performed on cryopreserved PBMCs isolated longitudinally just before, during, and after ATI for PrEP Participant A and HSCT Participant B. Thawed PBMCs were stained with LIVE/DEAD Fixable Aqua Dead Cell Stain Kit (ThermoFisher Scientific), Brilliant Violet 711-conjugated anti-CD4 (SK3) (BD Biosciences), Brilliant Violet 650-conjugated anti-CD3 (SP34-2) (BD Biosciences), allophycocyanin (APC)-conjugated anti-CD38 (HB7) (BD Bioscience), PE-conjugated anti-CD30 (BERH8) (BD Biosciences), APC-H7-conjugated anti-HLA-DR (L243) (BD Bioscience), CD8 Alexa Fluor 700-conjugated anti-CD8 (RPA-T8) (BD Bioscience), BV786-conjugated anti-CD16 (3G8) (BD Bioscience), PE-Cy7-conjugated anti-CD107a (H4A3) (BD Bioscience), FITC-conjugated anti-CD56 (NCAM 16.2) (BD Bioscience), and PerCP-conjugated anti-CD69 (L78) (BD Bioscience). Cells were then analyzed on a BD LSRII flow cytometer (BD Biosciences). Single stained beads (Life Technologies) were used for compensation, and fluorescence minus one (FMO) controls were used to set gates. Data for phenotyping were acquired on all events and analyzed in FlowJo V10 (TreeStar). Examples of gating strategies are shown in **S1 Fig**.

CD4+ and CD8+ T cell phenotypes were further characterized on live cells for PrEP Participant B by flow cytometry after exclusion of dead cells (Fixable Aqua dye, Molecular Probes) using the following fluorescently labeled antibodies: CD3 BV650 clone SK7, CD4 BV711 clone OKT4, CCR7 BV785 clone G043H7, CD45RA APC-Cy7 clone H100, Tbet PE clone 4B10, Eomesodermin PE-e610, PD-1 BV421 or BV605 clone EH12.2H7, CD160 PE-Cy7 clone BY55, Ki-67 FITC clone Ki-67, Granzyme B FITC clone GB11, Perforin PE-Cy7 clone B-D48 (all Biolegend), and CD8α APC-R700 clone RPA-T8 (BD Biosciences). Positive gates for activation markers and effector cell transcription factors were drawn based on expression of these markers in peripheral blood naive CD8+ T cells isolated from an HIV-uninfected donor. To limit experimental variability, flow cytometry was performed on the same day.

### Flow cytometric analysis of HIV-1–specific immune responses by intracellular cytokine staining

After thawing, PBMCs were incubated at 37°C overnight at a concentration of 2 × 10^6^ cells/mL in RPMI medium containing 10% FBS and penicillin/streptomycin. The next day, 1 × 10^6^ cells were stimulated with Gag pooled peptides (final concentration 1 μg/mL in DMSO provided by the NIH AIDS Reagent Program, Division of AIDS, NIAID, NIH: HIV-1 Con B Gag Peptide Pool cat #12425), for 6 hours in the presence of brefeldin A and monensin (BD). The percentage of CD8+ or CD4+ T cells producing interferon gamma (PE-dazzle, clone 4S.B3, Biolegend), tumor necrosis factor alpha (Alexa fluor 700, clone mAb11, eBioscience) or interleukin-2 (BV785, clone MQ1-17H12, Biolegend), or degranulation as detected by CD107a expression (APC, clone H4A3, Biolegend), was measured by flow cytometry, and the frequency of positive cells was determined after subtraction of the frequency measured in wells incubated with DMSO alone. Examples of gating strategies are shown in **S2 Fig**.

### Data analysis and mathematical modeling

Graphical analyses were performed using Prism v.6 (GraphPad software). Mathematical modeling of reservoir size, rebound rate, and probability of cure were performed using our previously described methods [35]. A summary of these methods and the input variables used is provided in **S1 Text**.

## Results

### Reduced HIV reservoir seeding following very early initiation of ART

Participant A, a 54-year-old male, was HIV-uninfected at 2 PrEP pre-enrollment visits but continued to have ongoing sexual risk for HIV infection. He then initiated tenofovir/emtricitabine PrEP and usage was confirmed by testing drug levels. Seven days after PrEP initiation, results from the baseline (day 0 of PrEP) visit revealed a plasma HIV RNA level of 220 copies/mL (Abbott RealTime HIV-1, lower limit of detection [LLD] <40 copies/mL), and 69 copies/mL by the Single-Copy Assay (LLD 1 copy/mL); 4th generation EIA (Abbott) and rapid HIV-antibody (Clearview HIV 1/2 Stat-Pak) tests were negative. Based upon these results (and a negative pooled HIV RNA, 4th generation EIA, and rapid HIV-antibody tests at 2 pre-enrollment visits 21 and 13 days prior), it was determined that he had been in the transition from the eclipse phase to Fiebig stage I HIV infection (estimated 10 days after HIV infection [12,13]) at the time of initiating PrEP. PrEP was substituted with a 4-drug ART regimen (darunavir/ritonavir/raltegravir/tenofovir/emtricitabine) 7 days after the initiation of PrEP. This ART regimen was chosen due to concern about the potential for the development of drug resistance to tenofovir/emtricitabine. The participant was asymptomatic at the time. HIV western blot assays (BioRad) were repeatedly indeterminate (p55 only) and became nonreactive at an estimated 130 days after time of infection. SGS from the plasma sample from the date when PrEP was initiated confirmed that the individual was infected with subtype B virus without known drug resistance mutations.

**Fig 1:**
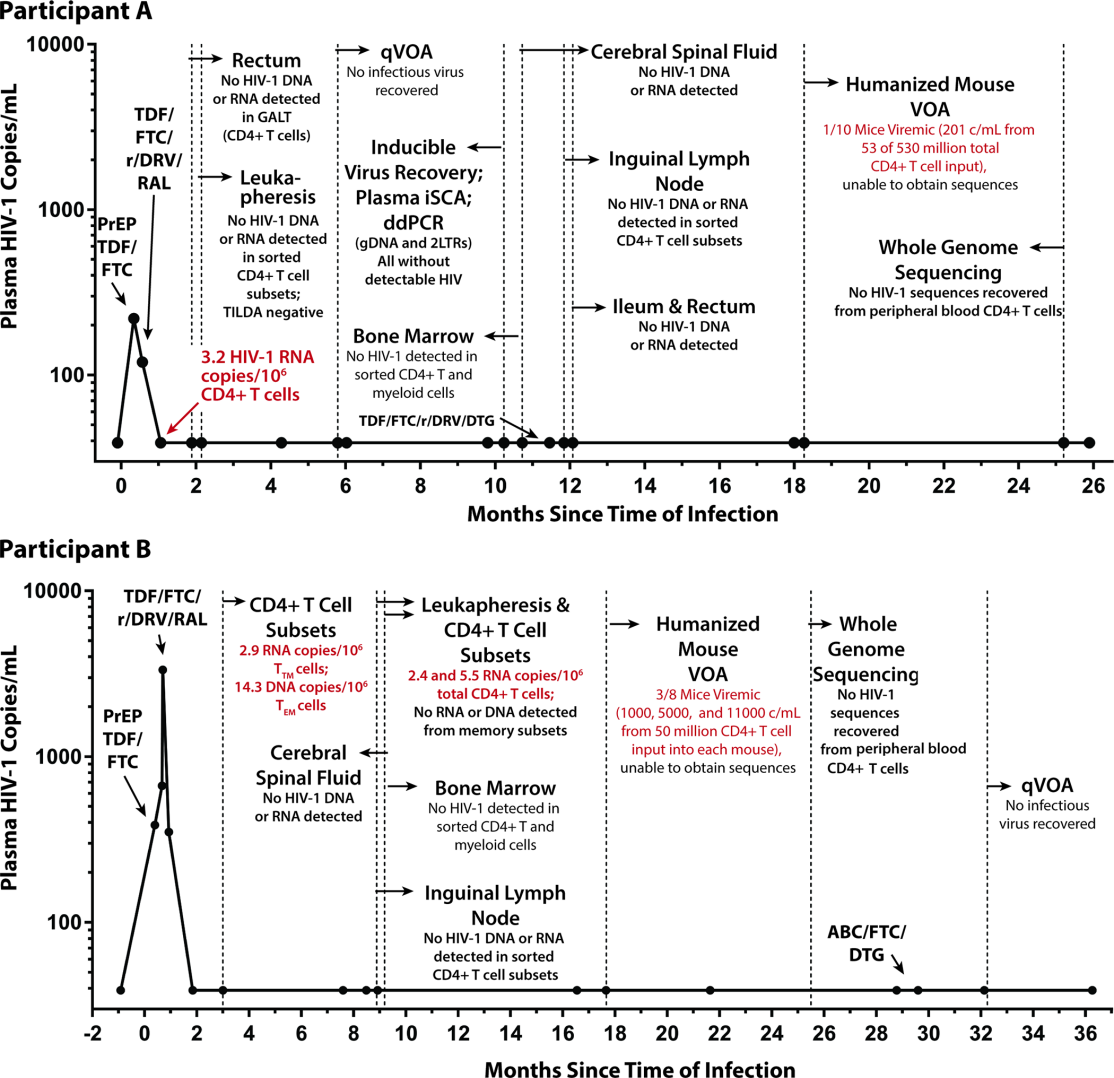
Extremely early ART reduces HIV reservoir seeding. No HIV was detected in colorectal and lymph node tissues, bone marrow, CSF, plasma, and very large numbers of PBMCs obtained longitudinally from Participant A (top panel) who started PrEP 10 days following HIV infection. In contrast, low-level HIV RNA and DNA were intermittently detected in PBMCs from Participant B (bottom panel) who initiated PrEP 12 days following infection prior to starting a 4-drug ART regimen. One of 10 humanized mice given 53 million input cells from Participant A developed low-level HIV RNA in plasma (201 copies/mL), but sequencing of HIV from plasma was unsuccessful. Abbreviations: ABC, abacavir; CSF, cerebral spinal fluid; ddPCR, droplet digital PCR; DTG, dolutegravir; FTC, emtricitabine; GALT, gut-associated lymphoid tissue; iSCA, HIV integrase single copy plasma RNA assay; PBMC, peripheral blood mononuclear cell; PrEP, pre-exposure prophylaxis; qVOA, quantitative viral outgrowth assay; RAL, raltegravir; r/DRV, ritonavir-boosted darunavir; TDF, tenofovir; TILDA, Tat/Rev Induced Limiting Dilution Assay; VOA, viral outgrowth assay.

Plasma HIV RNA levels subsequently declined following PrEP and combination ART initiation: 220 copies/mL (PrEP baseline), 120 copies/mL (7 days after initiation of PrEP), and <40 copies/mL but detectable (estimated 22 days after starting PrEP). All subsequent plasma HIV RNA levels were undetectable. Low-level cell-associated HIV RNA (3.2 copies/million CD4+ T cells) was detected 32 days after infection. However, sorted rectal CD4+ T cells were negative for HIV RNA and DNA (collected 1.9 months after infection), and leukapheresis-collected PBMCs enriched for total CD4+ T cells and sorted CD4+ T cell subsets (T_N_, T_CM_, T_TM_, and T_EM_) were negative for cellular HIV RNA, total HIV DNA (confirmed in 2 independent laboratories), integrated HIV DNA, and 2-LTR circles (collected 2.1 months after infection). Over the following 2 years, no further HIV (nucleic acid, viral outgrowth, total virus recovery, or whole genome sequences) could be detected, despite sampling from ileum, rectum, lymph nodes, bone marrow, CSF, and circulating CD4+ T cell subsets. A detailed timeline of clinical viral load measurements and results of longitudinal HIV-1 reservoir assays are shown in **Fig 1** and **Table 1**. Chemokine receptor 5 (CCR5) genotyping revealed that the individual did not carry any CCR5 Δ32 mutations, and HLA typing revealed that he was HLAB5701 negative. We estimated that greater than 1 billion CD4+ T cells were eventually examined without any evidence of HIV infection.

**Table 1:** HIV-1 assay results from Participant A longitudinal pre-ATI blood and tissue sampling.

Lab/ Location	Days Post-ART	Sample Type	Assay Type	Result	Sample Volume Tested or LOD
UCSF	22	PBMC	HIV RNA/DNA	3.2 RNA c/10^6^ CD4 + T cells; DNA neg	2.8 DNA copies/10^6^ cells
UCSF	47	Rectum/GALT	HIV RNA/DNA	Neg	16.6 (DNA), 25.4 (RNA) copies/million cells
UCSF & U. Montreal	55	PBMC (sorted)^1^	HIV RNA/DNA	Neg	CD4+ T cell subsets (LODs 0.5 to 37.2 copies/million cells)
U. Montreal	55	PBMC	TILDA	Neg	736,000 CD4+ T cells
Hopkins (Siliciano)	166	PBMC	qVOA	Neg	40 million resting CD4+ T cells
UCSF	288, 301, 316, 756, 1034	PBMC	HIV RNA/DNA	Neg	0.7 to 4.6 DNA or RNA copies/million CD4+ T cell
Pittsburgh	301	PBMC	Total Inducible Virus Recovery	Neg	25 million CD4+ T cells
Pittsburgh	301	Plasma	Plasma iSCA	Neg	29 mL (<0.06 copies/mL)
Pittsburgh	301	PBMC	HIV DNA	Neg	4.4 million CD4+ T cells
UCSD	301	PBMC	ddPCR for HIV genomic DNA and 2LTR circles	Neg	2.4 copies/million cells
UCSF	316 HIV-1 D	Bone Marrow (sorted)^2^	HIV RNA/DNA	Neg	Total, CD4+ T cells, myeloid cells, HSPC: 0.2–97.5 copies/million cells
UCSF	316	CSF	HIV RNA	Neg	<40 copies/mL
		CSF Cell Pellet	HIV RNA/DNA	Neg	457 (DNA) 68 (RNA) copies/million cells
UCSF	350	Lymph Node (inguinal, sorted)^1^	HIV RNA/DNA	Neg	0.8 to 28.2 copies/million CD4+ T cell immune subset
UCSF	357	Rectum (colonoscopy)	HIV RNA/DNA	Neg	0.9 (DNA), 6.9 (RNA) copies/million CD4+ T cells
UCSF	357	Ileum (colonoscopy)	HIV RNA/DNA	Neg	1.9 (DNA), 4.1 (RNA) copies/million cells
Hopkins (Blankson)	545	PBMC	mVOA	1/10 mice viremic (201 c/mL plasma)^3^	530 million CD4+ T cells
Hopkins (Siliciano)	756	PBMC	Whole genome sequencing	Neg	15 million resting CD4+ T cells

^1^ DNA and RNA obtained from sorted CD4+ T cell immune subsets (T_N_, T_CM_, T_EM_, and T_TM_).

^2^ DNA and RNA obtained from sorted CD4+ T cells, common lymphoid progenitor cells, hematopoietic stem/progenitor cells, and myeloid cells.

^3^ Unable to verify result.

Abbreviations: ART, antiretroviral therapy; ATI, analytical treatment interruption; c/mL, copies/mL; CSF, cerebrospinal fluid; ddPCR, droplet digital PCR; GALT, gut-associated lymphoid tissue; HSPC, hematopoietic stem and progenitor cells; iSCA, integrase single copy assay; LOD, limit of detection; LTR, long-terminal repeat; Neg, negative; PBMC, peripheral blood mononuclear cell; qVOA, quantitative viral outgrowth assay; TILDA, Tat/Rev Induced Limiting Dilution Assay; UCSD, University of California San Diego; UCSF, University of California San Francisco; VOA, viral outgrowth assay.

PrEP Participant B, a 31-year-old male, was HIV-uninfected at a pre-enrollment visit 6 weeks before initiating tenofovir/emtricitabine PrEP but had ongoing sexual risk for HIV infection. On the date of initiation of PrEP his Clearview HIV 1/2 Stat-Pak, HIV-1 CMIA (Abbott Ag/Ab combo assay), HIV-1 IFA, western blot, and HIV 1/2 MultiSpot Rapid Test were all non-reactive. Six days after initiation of PrEP, results from the baseline (day 0 of PrEP) visit revealed a plasma HIV of 359 copies/mL and PrEP was discontinued. Based on these results and clinical information, it was determined that he was infected approximately 12 days prior to starting PrEP (Fiebig stage I). Eight days after starting PrEP, the plasma HIV-1 RNA level increased to 668 copies/mL. HIV genotyping was positive for the M184M/I resistance mutation (no mutation was detected by genotyping on PrEP day 0), and 3,343 copies/mL were measured 9 days after the initiation of PrEP. He started combination ART (darunavir/ritonavir/raltegravir/tenofovir/emtricitabine) 12 days after starting PrEP, with subsequent decline of HIV plasma RNA to 351 copies/mL and 39 copies/mL on post-PrEP days 16 and 44, respectively. All subsequent plasma HIV RNA levels were undetectable starting at post-infection day 91. He changed ART to tenofovir/emtricitabine/dolutegravir/rilpiverine 56 days after starting PrEP and then to abacavir/lamivudine/dolutegravir 29 months following initial PrEP (**Fig 1**). HIV antibody testing remained negative through day 219 after the initiation of PrEP, but a western blot was indeterminate (p24 weekly positive, all other bands negative) on day 258.

Three months following infection, Participant B had very low level detectable HIV-1 RNA (2.9 copies/10^6^ cell) and DNA (14.3 copies/10^6^ cells) in T_TM_ and T_EM_ CD4+ T cell subsets, respectively, and again 2.4 and 5.5 HIV RNA copies/10^6^ total CD4+ T cells approximately 9 months after infection. No HIV could be detected from CSF, bone marrow cells or CD4+ T cell subsets from a whole excised inguinal lymph node 8–9 months following infection. No definitive HIV outgrowth could be detected with a qVOA performed 32 months after infection utilizing 20 million input total CD4+ T cells. Very low levels of HIV-1 RNA (<50 copies/mL) were detected from several of the viral outgrowth assay (VOA) wells, but no increases were observed in RNA over time, and no bulk or single genome HIV-1 sequences could be obtained (see **Fig 1** and **Table 2**).

**Table 2:** HIV-1 assay results from PrEP Participant B longitudinal blood and tissue sampling.

Lab/ Location	Days Post-ART	Sample Type	Assay Type	Result	Sample Volume Tested or LOD
UCSF	91	PBMC (leukapheresis)	HIV RNA/DNA	14.3 DNA c/10^6^ CD4+ T_EM_; 2.9 RNA c/106 CD4+ T_TM_	1 to 5.7 (RNA) and 14 to 14.2 (DNA) copies/10^6^ cells
UCSF	266	PBMC	HIV RNA/DNA	2.4 RNA c/10^6^ total CD4+ T cells; DNA Neg	12.4 DNA copies/10^6^ cells
UCSF	271	PBMC^1^ (leukapheresis)	HIV RNA/DNA	5.5 RNA c/106 CD4+ T cells; DNA and RNA neg from immune subsets	1.6 to 7.6 (RNA) and 11.6 to 664 (DNA) copies/10^6^cells
UCSF	272	Lymph Node^1^ (inguinal, sorted)	HIV RNA/DNA	Neg	13.7 to 18302 (DNA) and 1.3 to 3.9 copies/10^6^cells
UCSF	279	Bone Marrow (sorted)^2^	HIV RNA/DNA	Neg	0.2 to 15.8 (RNA) and 10.5 to 83 (DNA) copies/10^6^ cells
UCSF	656	PBMC	HIV RNA/DNA	Neg	6.3 (RNA) and 21.9 (DNA) copies/10^6^ cells
Hopkins (Blankson)	523	PBMC (leukapheresis)	mVOA	3/8 mice viremic (1,000, 5,000, 11,000 c/mL plasma)	500 million CD4+ T cells
Hopkins (Siliciano)	761	PBMC (leukapheresis)	Whole Genome Sequencing	Neg	15 million resting CD4+ T cells
UCSF	966	PBMC (leukapheresis)	qVOA	Neg	30 million total CD4+ T cells

^1^ DNA and RNA obtained from sorted CD4+ T cell immune subsets (T_N_, T_CM_, T_EM_, and T_TM_).

^2^ DNA and RNA obtained from sorted CD4+ T cells, common lymphoid progenitor cells, hematopoietic stem/progenitor cells, and myeloid cells.

Abbreviations: ART, antiretroviral therapy; c/mL, copies/mL; GALT, gut-associated lymphoid tissue; LOD, limit of detection; Neg, negative; PBMC, peripheral blood mononuclear cells; PrEP, pre-exposure prophylaxis; qVOA, qVOA, quantitative viral outgrowth assay; UCSF, University of California San Francisco.

### mVOA

Given the very low level or lack of detectable HIV-1 from large numbers of cells from various tissues, large quantity plasma, or CSF, we performed a leukapheresis on both participants and used large numbers of CD4+ T cells for input into a previously reported mVOA [34]. We obtained 530 million total CD4+ T cells from Participant A approximately 18 months following infection on ART. One of 10 mice given 53 million input cells developed low-level HIV RNA in plasma (201 copies/mL) following in vivo activation with anti-CD3 antibody approximately 5.5 weeks after engraftment, but HIV was not detected in spleen tissue (<1 million spleen cells present). However, HIV-1 RNA and cDNA sequencing at an independent laboratory was unsuccessful from plasma and spleen cells, and true infection in cells from Participant A could not be verified. We obtained 500 million total CD4+ T cells from Participant B. Three of 8 mice developed detectable HIV RNA starting approximately 4.5 weeks following cell transfer (1,000; 5,000; and 11,000 copies/mL, respectively, from a total of 50 million CD4+ T cells per mouse).

### ATI

**Fig 2:**
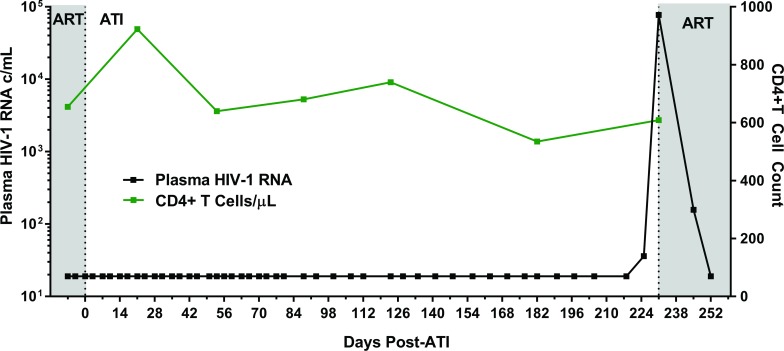
Summary timeline of plasma viral load and CD4+ T cell counts following analytical ART treatment interruption in Participant A. Abbreviations: ART, antiretroviral therapy; ATI, analytical treatment interruption; c/mL, copies/mL.

Following 34 months of continuous ART, Participant A provided informed consent to undergo a carefully monitored ATI (**Fig 2**). The patient remained clinically well with undetectable plasma viral load measurements through 225 days of observation post-interruption. On day 225, he had a detectable, low-level plasma HIV-1 RNA of 36 copies/mL. Repeat testing 6 days later (day 231) confirmed rebound with virus increasing to 77,397 copies/mL. He initiated therapy (tenofovir/emtricitabine/ritonavir/darunavir/dolutegravir) on post-ATI day 231 prior to receiving the confirmatory test. He then had viral loads of 158 and 19 copies RNA/mL on days 245 and 252, respectively. Subsequent plasma viral load testing revealed no detectable HIV RNA, and he has remained clinically well. Single genome sequences of a 1,190 base-pair region of HIV-1 Pol were monoclonal and identical to the PrEP baseline sequences. The level of intra-sequence diversity was extremely low at both time points (<0.02%) but distinct from the consensus ancestral subtype B sequence. HIV-1 envelope single genome (1,557 base-pair region) analysis at the time of recrudescence revealed 2 unique but highly related sequences; HIV envelope sequences were not obtained at PrEP baseline (**S3 Fig**).

### Estimates of HIV reservoir size following extremely early initiation of ART

We used a collection of mathematical modeling approaches (see **S1 Text**) to better understand the dynamics of reservoir seeding and rebound in these participants. For PrEP Participant A, the estimated latent reservoir size immediately before treatment interruption, based only on the observed time of rebound (226 days) [36] was 0.0020 [0.00045, 0.0063] infectious units per million cells (IUPM) (brackets give 95% credible intervals). With this estimated reservoir size (including uncertainty), about 1% of identical individuals (i.e., having similar restrictions in reservoir size) would be expected to achieve lifelong (>70 year) ART-free HIV remission. These results are consistent with estimates of reservoir size from various assays described in Fig 1 and Tables 1 and 2. Based on the viral load level at initial diagnosis and a calibrated model of reservoir seeding during acute infection [37], we estimate that the reservoir size prior to the initiation of ART was 0.02 IUPM, with about 1 log uncertainty in either direction. qVOA results on day 166 suggest that there is a 95% probability that the reservoir size is below 0.075 IUPM [38]. If we assume the positive outgrowth in the mVOA is a true positive signal, then there is a 95% probability that the reservoir size is below 0.0057 IUPM. If we assume the outgrowth was not real, then the central estimate for the reservoir size is 0.0020 IUPM (95% credible interval 0.00028–0.014 IUPM). Assuming there are around 10^11^ total body CD4+ T cells [39,40], these estimates collectively suggest there were only approximately a few hundred cells infected with replication-competent HIV provirus prior to treatment interruption. Separately, we estimated the exponential growth rate of viral load during rebound to be 1.3/day, very similar to that seen in the HSCT Boston participants [36] and near the central value seen in acute infection [41–43], but much higher than that seen in rebound following chronic infection (excluding the prolonged time off ART prior to first detection of HIV-1) [44–46].

### Surface marker expression and HIV-1–specific responses prior to and following ATI

**Fig 3:**
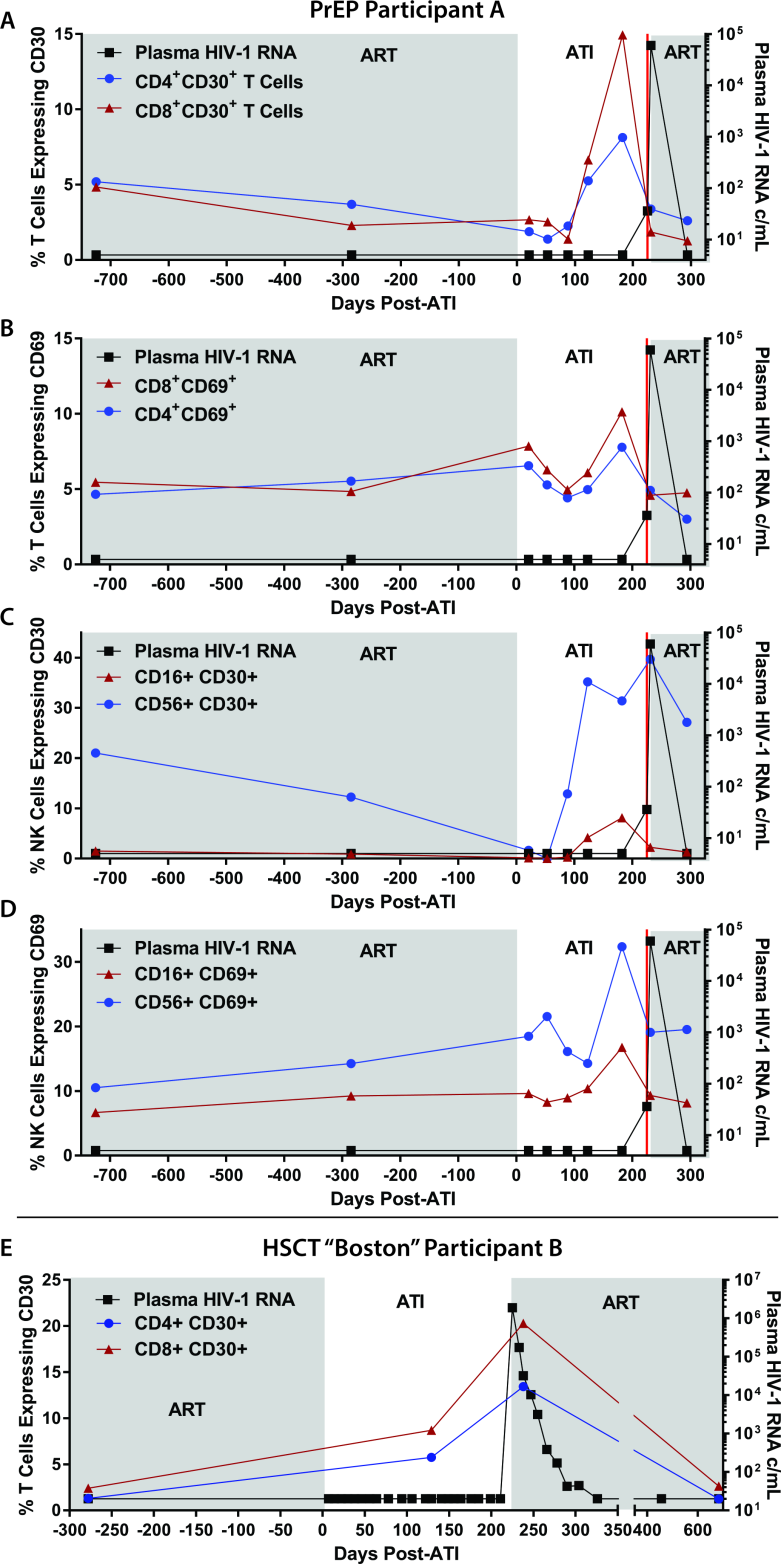
Changes in lymphocyte surface markers following ATI in PrEP Participant A and HSCT Participant B. The percentage of CD8+ and CD4+ T cells expressing CD30 and CD69 are shown in (A) and (B). The percentage of CD16+ and CD56+ NK cells expressing CD30 and CD69 are shown in (C) and (D). T cell expression of CD30 from the allogeneic HSCT recipient who also underwent ATI is shown in (E). Abbreviations: ATI, analytical treatment interruption; c/mL, copies/mL; HSCT, hematopoietic stem cell transplantation; NK, natural killer; PrEP, pre-exposure prophylaxis.

Flow cytometric characterization of surface markers of T and natural killer (NK) cell activation was performed on samples from PrEP Participant A before and during ATI and following HIV rebound in order to identify potential predictors of viral recrudescence. In addition, pre- and post-ATI samples were investigated from HSCT Participant B, an individual who lost detectable HIV-1 in blood and gut tissue following allogeneic HSCT for malignancy. Similar to PrEP Participant A, the HSCT participant experienced confirmed HIV rebound 225 days after interrupting ART and had similar exponential growth rate during recrudescence [16]. Interestingly, surface expression of CD30, a member of the tumor necrosis factor (TNF) super-receptor family and lymphoma tumor marker, increased on CD4+ and CD8+ T cells months prior to detectable plasma HIV in both of these participants (**Fig 3**). The frequency of CD30+ and CD69-expressing cells also increased on CD4+ and CD8+ T cells and CD56+ and CD16+ NK cells prior to viral recrudescence in PrEP Participant A. Overall, increases in the frequency of CD30-expressing cells appeared to be larger than those expressing CD69. However, no distinct patterns in lymphocyte HLA-DR/CD38 were observed in either participant, and only CD30 expression increased prior to rebound in the HSCT recipient.

Sufficient cells were available for further immunological phenotyping from PrEP Participant A, and we identified an increase in the frequency of eomesodermin expressing CD8+ T cells prior to viral rebound. However, no patterns emerged during ATI for expression of PD1, TIGIT, or Ki67. There were no changes in CD8 responses in CD107a expression, and no significant intracellular INF-gamma, TNF-alpha, or IL1 production was detected in response to overlapping HIV-1 gag peptide pools prior to, during, or following ATI.

## Discussion

We report 2 cases of extremely early HIV diagnosis and initiation of ART at the threshold when plasma viremia begins to expand exponentially (the end of the so-called “eclipse phase” and beginning of Fiebig stage I). These stages of HIV infection precede the time when acute HIV infection becomes clinically apparent and are theoretically the earliest time when ART can be initiated in an adult [12,13]. To the best of our knowledge, PrEP Participant A is the earliest documented case of adult HIV infection followed by immediate initiation of ART with the exception of successful post-exposure ART prophylaxis, and it would be very challenging to initiate therapy any earlier. Despite the complete or near-complete loss of detectable HIV in blood and a variety of tissues, HIV rebounded in this person 225 days following cessation of ART. Although identifying hyperacute infection is rare, our group has previously reported that 15.6% of patients referred to our HIV clinic for newly diagnosed infection had detectable plasma HIV-1 RNA but negative HIV-specific antibody test results [47]. These data include individuals taking part in a rapid treatment initiation study and may overestimate the incidence of acute infection identified during Fiebig stage I. Excluding participants in this study, 6.3% of individuals presented with positive plasma HIV-1 RNA and no detectable HIV-specific antibodies at the time of diagnosis [47].

The delayed timing of viral rebound in PrEP Participant A was similar to that observed in an HIV-infected individual who lost detectable HIV in blood and tissue following allogeneic HSCT (Boston Participant B). In each case, modeling predicted a residual HIV replication-competent reservoir of perhaps hundreds of infected CD4+ T cells throughout the body, which explains the lack of detectable HIV from blood or tissues despite massive sampling. Overall, allogeneic HSCT and extremely early ART initiation appear to have similar long-term effects on reducing both the residual HIV reservoir and immune responses. Nonetheless, small numbers of latently infected cells likely persisted in these individuals and became activated, leading to HIV rebound in the absence of ART. Modeling of these types of participants leads to wide confidence intervals, which are likely due to the varying size of the reservoir in different patients, uncertainty around model parameters, and the stochastic nature of reactivation of latently infected cells in vivo. It is also possible that a non-CD4+ T cell source of HIV persistence contributed the viral rebound; a majority of cells tested for persistence in blood and tissue were CD4+ lymphocytes. Of note, the median time to viral rebound in 8 Thai individuals treated with ART during later phases of Fiebig stage I was recently reported to be 26 days [48]. Although sample size is limited, these and our data suggest that differences in initiation of ART time of just a few days during Fiebig stage I infection may have a noticeable impact on the duration of ART-free remission.

After this delay, the rapid HIV rebound dynamics in PrEP Participant A were again similar to those observed in HSCT Participant B [16], consistent with rapid exponential growth seen with primary infection. Unlike the HSCT participant, however, the PrEP recipient was asymptomatic and the peak viral load may have been mitigated by the earlier reinitiation of ART. The observed rapid rebound kinetics also differ from those in individuals who achieved post-treatment HIV-1 control following early ART initiation [49].The rapid rebound kinetics and lack of post-treatment control observed in this study are likely secondary to the lack of HIV-specific immunity since exponential growth is lower in the setting of withdrawal of ART initiated during chronic infection when HIV-specific immunity is present. Concomitant immune-modifying therapies may be necessary in order to achieve ART-free HIV control in very-early treated individuals.

PrEP Participant B initiated ART later than PrEP Participant A and had higher levels of viremia at his baseline visit. In many ways, he is similar to the “Mississippi baby” [6,7] in that he started ART with moderate levels of viremia and subsequently had a difficult-to-detect reservoir. Individuals who initiate therapy during the earliest stages of infection will almost certainly need other “curative” interventions before ART might be interrupted with the expectation that a viral relapse will be avoided. It is also possible that higher peak plasma HIV RNA levels and subsequent low-level viral reservoir detection in samples from PrEP Participant B were a result of the appearance of an emtricitabine-resistant associated mutation and exposure to a single active antiretroviral drug during initial PrEP.

It is likely that there are rare individuals who start PrEP during the true eclipse phase, when HIV has the potential to establish a long-lived reservoir but before the development of detectable HIV in blood or tissues. Detecting a potential “cure” in such a setting will be challenging but such efforts are ongoing. Of note, non-human primate studies have demonstrated that, while ART initiated within 48 hours of SIV challenge is able to prevent infection [50–52], ART started 3 days following SIV infection leads to viral rebound following cessation of therapy [53]. As a result, the "curative window" between infection and the potential to abort infection after exposure is likely very small. It is also possible that there may be individuals infected just before the start of PrEP, but in whom infection would not be known for some time until they stop ART or until a 2-drug regimen is unable to suppress the virus.

Our study is one of the first to incorporate an mVOA to measure potential HIV persistence following an intervention that leads to loss of detectable HIV in blood and various tissues. One mouse became viremic (at low levels) and 3 mice became viremic following receipt of cells from PrEP Participants A and B, respectively. This positive finding with PrEP Participant A's PBMCs may be the only evidence that this individual had persistent HIV prior to ART interruption. Unfortunately, sequence verification of plasma and splenic HIV in mice from both PrEP participants could not confirm definitive viral outgrowth from participant cells. Sample volumes are limited in murine models of HIV infection and were exhausted during testing. Nonetheless, 2 published studies suggest that mVOAs may be more sensitive than traditional outgrowth assays to detect replication-competent HIV in individuals with undetectable HIV-1 DNA or RNA by traditional means [34,54]. If this is the case, our study suggests that sampling of hundreds of millions of PBMCs may, at times, be more sensitive than tissue-based studies for the detection of residual HIV infection since a much larger number of cells can be interrogated. Further studies comparing mVOAs with traditional ex vivo co-culture assays utilizing rigorous positive and negative controls are certainly warranted.

A major emphasis of HIV curative science has been to identify potential markers or correlates of HIV rebound before or after treatment cessation. CD30, a member of the TNF receptor superfamily, is expressed on very small percentages of lymphocytes and myeloid cells but is dramatically upregulated on Hodgkin and other lymphoma cells [55–59]. CD30 has been implicated in the activation, proliferation, and cell death of selected cell populations [55,57,60,61]; and infections with human T-cell lymphotropic virus (HTLV), Epstein-Barr virus (EBV), and poxviruses can lead to increases in CD30 expression [57,62,63]. In addition, increases in the plasma concentration of the soluble form of CD30 have been associated with HIV disease progression prior to ART initiation [57,58,60,64–71]. Although anecdotal, our data in PrEP Participant A and the HSCT participant suggest that upregulation of CD30 may occur prior to detectable plasma HIV-1 RNA. Furthermore, given a similar but somewhat less pronounced pre-rebound expression pattern of CD69 in the PrEP Participant A, it is possible that these markers represent more global lymphocyte activation, proliferation, or stress responses that are detectable prior to viral recrudescence. Further investigation of CD30 and related cell-surface markers as potential predictors of HIV rebound is urgently needed.

These cases also suggest that PrEP programs should test individuals for HIV using an HIV test with the narrowest window period possible, just before initiating PrEP. If cost permits and in settings with a high incidence of acute HIV (e.g., STI clinics), it is reasonable to test with a plasma HIV RNA before PrEP initiation and when reinitiating PrEP after an interruption. Fourth generation combination p24 antigen/antibody tests will identify most patients with acute HIV but miss those in the earliest Fiebig stages, such as those presented in this analysis. Plasma HIV RNA testing should also be considered when reinitiating PrEP after interruption [9]. PrEP programs should recommend an immediate switch to conventional ART if an individual is found to be newly HIV-positive. Potential benefits of such programmatic changes include (1) lower rates of missed acute HIV diagnoses, (2) decreased acquired drug resistance to tenofovir/emtricitabine, (3) individual clinical benefit [72], and (4) fewer subsequent transmission events [73].

This study was limited by its small sample size. The identification of individuals treated during hyperacute infection is rare given the rapid increase in plasma HIV-1 RNA during the early phase of Fiebig stage I infection and the fact that hyperacute infection may be asymptomatic. The limited number of individuals included in the analysis make it difficult to draw definitive conclusions about the impact of very early ART on restricting HIV reservoir size or prolonging ART-free remission. In addition, a larger number of individuals are required to validate potential cell-surface markers of HIV persistence or predictors of HIV rebound. Despite the challenges with including very early treated individuals in prospective studies, these cases provide valuable information as to the rapidity of seeding of the HIV reservoir and the impact of extremely early ART on HIV persistence.

In summary, we report 2 cases of extremely early initiation of prophylactic ART immediately following the “eclipse phase” in Fiebig stage I (at approximately 10 days of HIV infection). A very small residual HIV reservoir size was observed in these participants who started very early PrEP and subsequently converted to full ART. In 1 individual, we observed a prolonged period of ART-free remission similar in duration to the allogeneic HSCT Boston Participant B. However, although HIV persisted indefinitely in both of these PrEP cases, a continuum may exist across PrEP, post-exposure prophylaxis and curative early ART strategies. Further investigation in larger cohorts of individuals treated extremely early following HIV infection is warranted.

## Supporting information

S1 STROBE Checklist (PDF)

S1 Fig Gating strategy for lymphocyte activation and tumor marker expression. (PDF)

S2 Fig Gating strategy for CD4+ and CD8+ T cell markers of immune checkpoint and exhaustion. (PDF)

S3 Fig Maximum likelihood phylogenetic trees of HIV-1 Pol and Env single genome sequences. (PDF)

S1 Text Mathematical estimates of HIV reservoir size. (PDF)

S1 Protocol Analytical Treatment Interruption (ATI) Protocol. (PDF)

## Abbreviations

ART: antiretroviral therapy
ATI: analytical treatment interruption
CCR5: chemokine receptor 5
CSF: cerebral spinal fluid
ddPCR: droplet digital PCR
GALT: gut-associated lymphoid tissue
HSCT: hematopoietic stem cell transplantation
IUPM: infectious units per million cells
LLD: lower limit of detection
LOD: limit of detection
MSM: men who have sex with men
mVOA: murine viral outgrowth assay
NK: natural killer
PBMC: peripheral blood mononuclear cell
PrEP: pre-exposure prophylaxis
qVOA: quantitative viral outgrowth assay
SGS: single-genome sequencing
TILDA: Tat/Rev Induced Limiting Dilution Assay
TNF: tumor necrosis factor
UCSD: University of California San Diego
UCSF: University of California San Francisco
VOA: viral outgrowth assay
