# Supplementary material for: HIV-1 persistence following extremely early initiation of antiretroviral therapy (ART) during acute HIV-1 infection: An observational study
Source: PLoS Med. 2017 Nov 7;14(11):e1002417. doi: 10.1371/journal.pmed.1002417 (PMC5675377; doi:10.1371/journal.pmed.1002417)
Supplement: S1 STROBE Checklist — (PDF) [file pmed.1002417.s001.pdf]

STROBE Statement—Checklist of items that should be included in reports of ***cohort studies***

	Item No	Recommendation
**Title and abstract**	1	We have provided in the abstract an informative and balanced summary of what was done and what was found
**Introduction**		
Background/rationale	2	The scientific background and rationale for the investigation being reported has been explained
Objectives	3	Specific objectives have been explained
**Methods**		
Study design	4	Key elements of study design are presented early in the paper
Setting	5	The setting and locations, and data collection have been provided
Participants	6	The eligibility criteria and the sources and methods of selection of participants have been provided. Follow-up methods have been provided.
Variables	7	Outcomes, exposures, predictors, potential confounders, and effect modifiers have been provided.
Data sources/ measurement	8	*S*ources of data and details of methods of assessment (measurement) have been provided
Bias	9	We discuss potential sources of bias
Study size	10	The study size rationale has been provided
Quantitative variables	11	N/A
Statistical methods	12	Statistical methods have been described
**Results**		
Participants	13	Numbers of individuals at each stage of study have been reported
Descriptive data	14	Characteristics of study participants have been provided when able as well as exposure information
		Follow-up time has been summarized
Outcome data	15	Numbers of outcome events have been reported
Main results	16	All results generated from these case reports have been provided in addition to confidence intervals and estimates when applicable
Other analyses	17	All analyses have been reported that have been performed and are pertinent to this study
**Discussion**		
Key results	18	Key results have been summarized with reference to study objectives
Limitations	19	Limitations of the study have been discussed
Interpretation	20	A cautious overall interpretation of results have been provided considering objectives, limitations, multiplicity of analyses, results from similar studies, and other relevant evidence
Generalisability	21	Generalisability of study results have been discussed
**Other information**		
Funding	22	Sources of funding and the roles of the funders for the present study have been provided
