# Supplementary material for: HIV-1 persistence following extremely early initiation of antiretroviral therapy (ART) during acute HIV-1 infection: An observational study
Source: PLoS Med. 2017 Nov 7;14(11):e1002417. doi: 10.1371/journal.pmed.1002417 (PMC5675377; doi:10.1371/journal.pmed.1002417)
Supplement: S1 Fig — (PDF) [file pmed.1002417.s002.pdf]

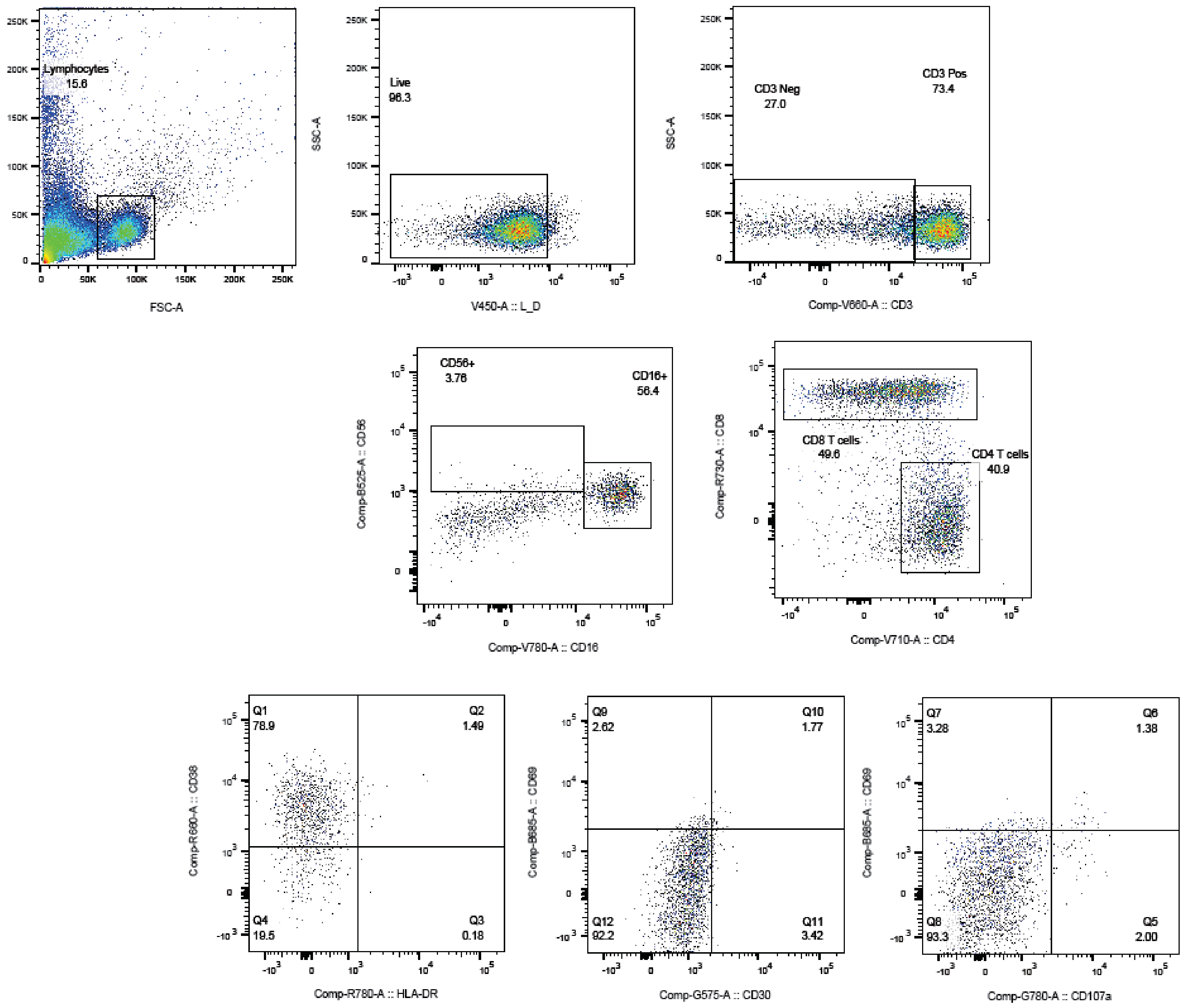

Fig S1. Example gating method for CD4+ lymphocyte expression of CD30 and CD69 during analytical treatment interruption.
