# Supplementary material for: HIV-1 persistence following extremely early initiation of antiretroviral therapy (ART) during acute HIV-1 infection: An observational study
Source: PLoS Med. 2017 Nov 7;14(11):e1002417. doi: 10.1371/journal.pmed.1002417 (PMC5675377; doi:10.1371/journal.pmed.1002417)
Supplement: S2 Fig — (PDF) [file pmed.1002417.s003.pdf]

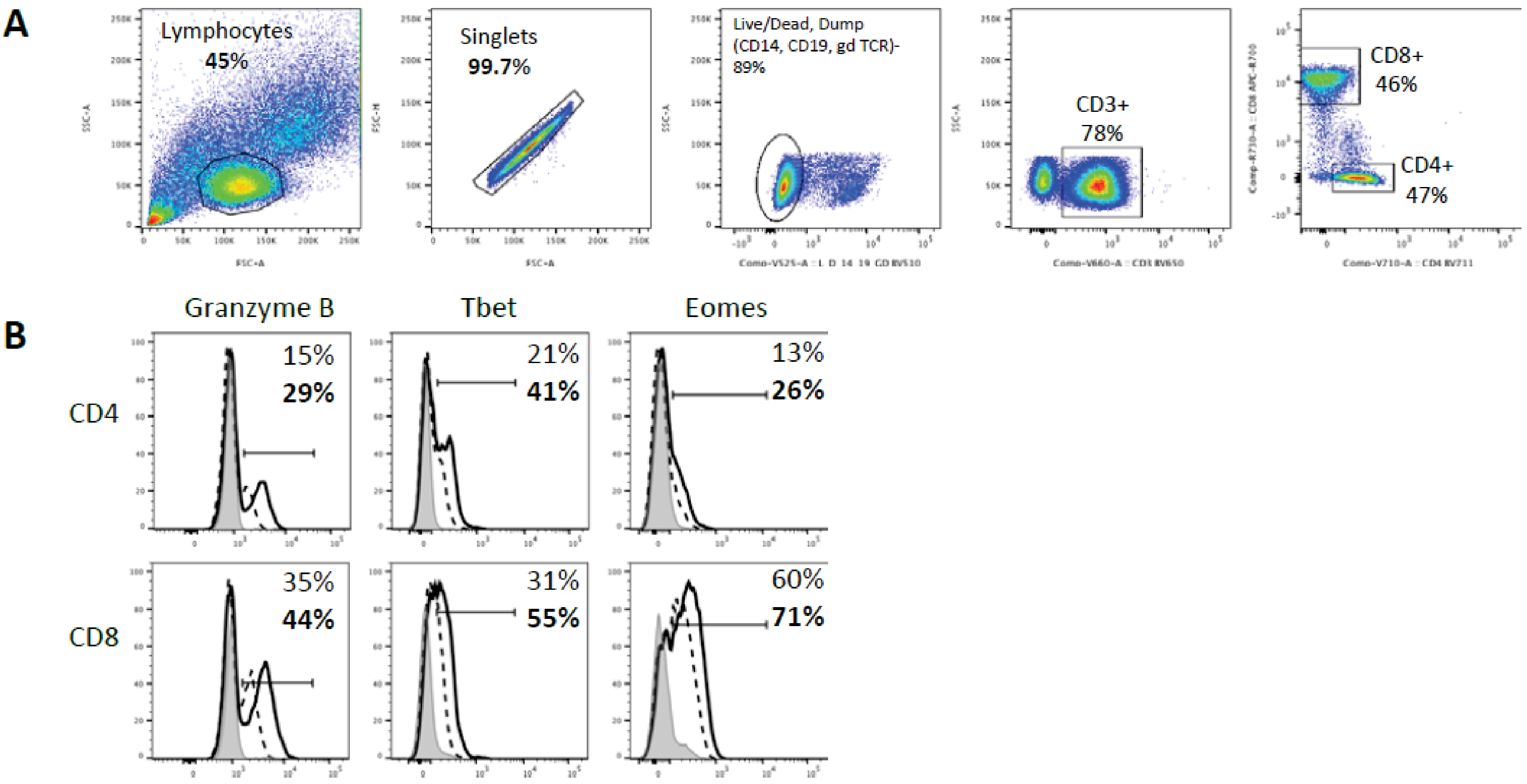

Fig S2. Gating strategy for CD4+ and CD8+ T cell markers of immune checkpoint and exhaustion. **A.** CD4+ and CD8+ T cell parent gates were analyzed by flow cytometry using the following gating strategy: lymphocytes, singlets, live cells staining negative for dump gate markers (CD14, CD19, gamma delta TCR), CD3+, CD4+ or CD8+. **B.** Percent of CD4+ (top row) and CD8+ (bottom row) T cells expressing Granzyme B, Tbet and Eomesodermin (Eomes) at day 53 (dashed line) and day 123 (solid line) post-analytic treatment interruption. Naïve (CD45RA+CCR7+) CD4+ or CD8+ population shown in grey for reference.
