# Supplementary material for: HIV-1 persistence following extremely early initiation of antiretroviral therapy (ART) during acute HIV-1 infection: An observational study
Source: PLoS Med. 2017 Nov 7;14(11):e1002417. doi: 10.1371/journal.pmed.1002417 (PMC5675377; doi:10.1371/journal.pmed.1002417)
Supplement: S3 Fig — (PDF) [file pmed.1002417.s004.pdf]

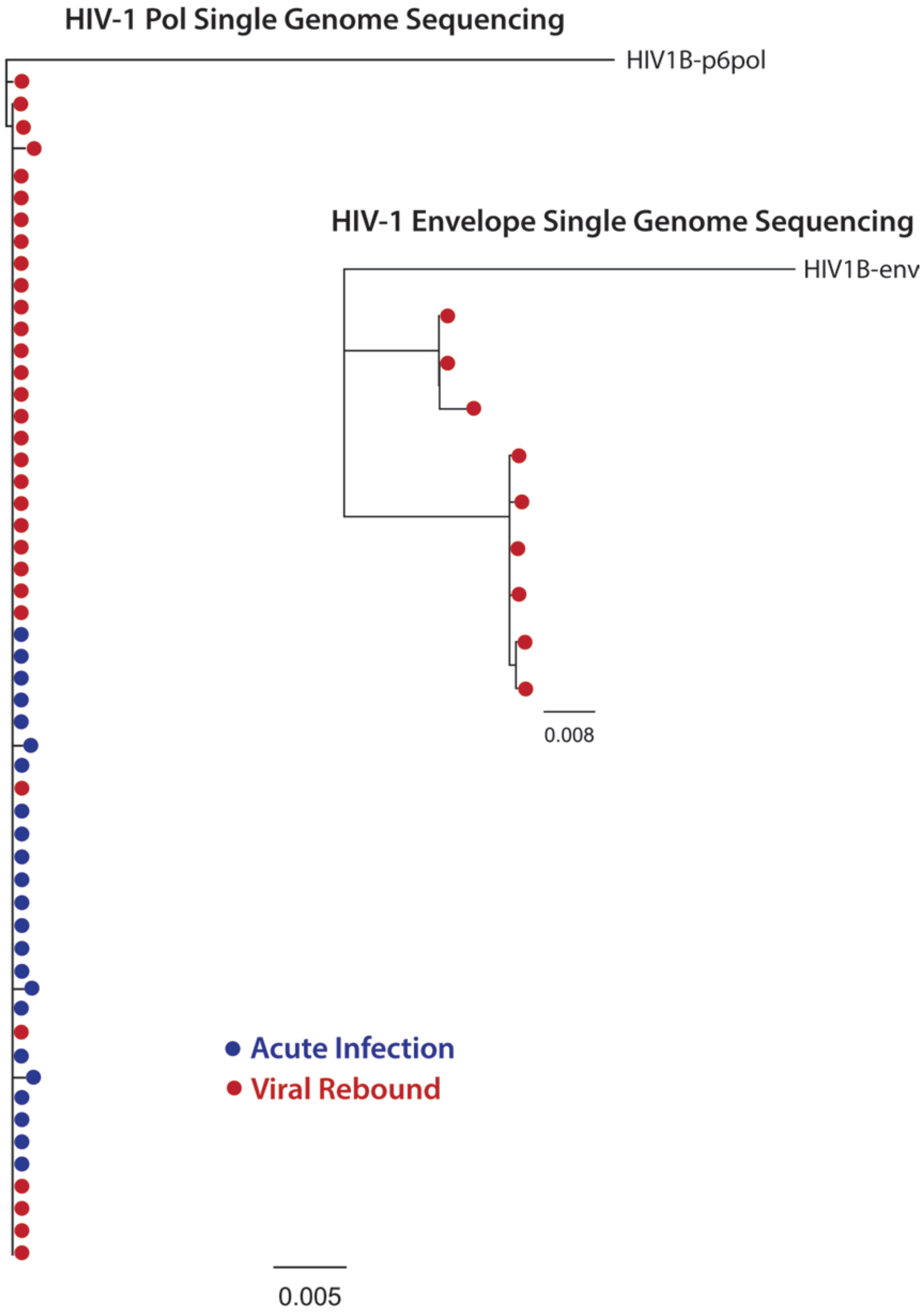

Fig S3. Maximum likelihood phylogenetic trees of HIV-1 Pol and Env single genome sequences obtained from PrEP participant B plasma RNA.
