# Supplementary material for: HIV-1 persistence following extremely early initiation of antiretroviral therapy (ART) during acute HIV-1 infection: An observational study
Source: PLoS Med. 2017 Nov 7;14(11):e1002417. doi: 10.1371/journal.pmed.1002417 (PMC5675377; doi:10.1371/journal.pmed.1002417)
Supplement: S1 Text — (PDF) [file pmed.1002417.s005.pdf]

Mathematical estimates of HIV reservoir size

Supplementary Methods

Estimating reservoir seeding pre-ART

We use a method described in our previous paper (SI Text X of that Hill et al [1]), which uses a calibrated model of reservoir seeding during acute infection based on the work of Archin et al [2].

To model the trajectory of infection pre-ART, we assume that viral load grew exponentially from an initial value v_0 to the detected value v_d, at a rate r. Since each participant was started ART relatively early, we believe the assumption of exponential growth is reasonable. As long as $v_0 << v_d$, (likely given that infection is started from a small number of virions), the value of v_d does not influence the results (we chose 10^{-3}). We assume that r = 1.1/day, based on the population-level mean observed in acute infection by Ribeiro et al [3]. We also assumed that CD4 levels had not yet significantly declined from baseline.

Note that when applying this model in the context of infection during PrEP, we assume that the presence of PrEP does not influence the probability that any new infection will result in latency.

For Participant A, the final viral load value before ART (PrEP) was initiated was 220 copies/mL. The estimated reservoir size is 0.02 IUPM, with uncertainty of up to 1 log in either direction (based on Archin et al [2]).

For Participant B, the viral load trajectory was more complicated as PrEP was started but only taken intermittently before ART was eventually initiated. We calculated total reservoir seeding by assuming different viral growth rates in different intervals: r = 1.1/day pre ART (until day 0 of PrEP when viral load was 359 copies/mL), then no new infections during PrEP from days 0 to 6, then viral load growing with rate r = 1.6/day between days 6 and 13 when PrEP was stopped and ART was not yet started. The latter r value was calculated using the difference in viral load of 668 to 3343 between days 8 and 9. The estimated reservoir size is 0.5 IUPM.
Note that the time of treatment initiation, and the observed reservoir sizes, are smaller in the participants of this study than in the study from which this model is calibrated, and so estimates may be outside the range of highest accuracy.

Estimating reservoir size from assay measurements during ART

We use the method described in our previous paper [4] and implemented in the online application IUPMStats (available at http://silicianolab.johnshopkins.edu/). To estimate the frequency of infected cells from limiting dilution outgrowth assays (done either in cell culture, qVOA, or in the humanized mouse model, hmVOA). This calculation assumes that the cells used the assay were randomly sampled from the much larger pool in the participant's body, that the assay is 100% sensitive and specific, and that the probability that any cell harboring HIV-provirus reactivates is independent of the presence of other infected cells in the same assay. Note that due to the expected extremely low frequency of infection in these participants, assays were often not done in a dilution series, but at a single dilution level. However, based on the low frequency of observing outgrowth, we believe it is safe to assume that this level represents limiting dilution, and therefore expect our calculations of infection frequency to be robust.

Estimates are reported as the median and 95% central credible intervals for the posterior estimate for the infection frequency.

For Participant A, the largest cell input to a laboratory assay occurred 6 months after ART was initiated (and ~2 years, 4 months before ART was interrupted). At this time, no inducible virus by qVOA [5] was found in a sample of 40 million resting CD4 T cells. This suggests there is a 95% probability that the reservoir size is below 0.075 IUPM (median of posterior is 0.017 IUPM).

The humanized mouse outgrowth assay was conducted 1.5 years after initiation of ART in Participant A (and ~1 year, 4 months before ART was interrupted). 53 million resting CD4 T cells were put into each of 10 mice (a total of 530 million cells). One mouse developed detectable HIV in the plasma, but this could not be verified by sequencing, making the output of this assay uncertain. Therefore, we considered both cases. If the positive outgrowth in one mouse is *ignored*, then there is a 95% probability that the reservoir is below 0.0057 IUPM (central estimate 0.0013). If we *include* the positive outgrowth in 1 out of 10 of the mice, then the central estimate for the reservoir size is 0.0020 (95% credible interval 0.00028 - 0.014 IUPM).

Estimating reservoir size from rebound time following ART-cessation

We use the method described in our previous paper [1], which uses a Bayesian framework to estimate a posterior distribution for the reservoir size at the time of ART-interruption, given an observation of rebound time. It uses the relationship between reservoir size and rebound time predicted by a mathematical model [6] as a likelihood function, and may optionally take assay input from the time of interruption as a prior.

For Participant A, rebound was observed 226 days following ART interruption. Using an uninformative prior on reservoir size, this rebound time suggests a reservoir size of 0.0020 IUPM (95% central credible interval of [0.00045, 0.0063]). Assuming a total body level of ~10^{11} resting CD4 T cells (based on a plasma volume of ~3L containing 2% of total body CD4 T cells at ~1000 cells/uL), this corresponds to around 200 residual infected cells at the time of ART interruption.

Including as a prior the fact that either 40 million (qVOA) or 530 million (hmVOA) cells tested negative for viral outgrowth does not really change this estimate.

Estimating probability of cure given reservoir size

We use our previously developed mathematical model [6] to estimate the probability of cure for a given reservoir size. To estimate the chance of cure in a hypothetical cohort of participants identical to Participant A, taking into account our uncertainty about pre-interruption reservoir size in this participant, we integrate the model prediction over the entire posterior distribution for Participant A’s reservoir size (which was estimated based on the rebound time, see above). We estimate a 1% probability of cure.

Estimating growth rate of viral rebound

We assume that viral load grows exponentially between the first and second detectable values during rebound.

For Participant A, viral load at Day 225 post-interruption was 36 copies/mL, and then was 77,397 copies/mL at Day 231, leading to an estimate of r = 1.3/day.

If the rate of viral load increase had already started to slow by Day 231, then the true early growth rate would be even higher.

References

1. Hill AL, Rosenbloom DIS, Goldstein E, Hanhauser E, Kuritzkes DR, Siliciano RF, et al. Real-Time Predictions of Reservoir Size and Rebound Time during Antiretroviral Therapy Interruption Trials for HIV. PLOS Pathog. 2016;12: e1005535. doi:10.1371/journal.ppat.1005535

2. Archin NM, Vaidya NK, Kuruc JD, Liberty AL, Wiegand A, Kearney MF, et al. Immediate antiviral therapy appears to restrict resting CD4+ cell HIV-1 infection without accelerating the decay of latent infection. Proc Natl Acad Sci U S A. 2012;109: 9523–9528. doi:10.1073/pnas.1120248109

3. Ribeiro RM, Qin L, Chavez LL, Li D, Self SG, Perelson AS. Estimation of the Initial Viral Growth Rate and Basic Reproductive Number during Acute HIV-1 Infection. J Virol. 2010;84: 6096–6102. doi:10.1128/JVI.00127-10

4. Rosenbloom DIS, Elliott O, Hill AL, Henrich TJ, Siliciano JM, Siliciano RF. Designing and Interpreting Limiting Dilution Assays: General Principles and Applications to the Latent Reservoir for Human Immunodeficiency Virus-1. Open Forum Infect Dis. 2015;2: ofv123. doi:10.1093/ofid/ofv123

5. Laird GM, Rosenbloom DI, Lai J, Siliciano RF, Siliciano JD. Measuring the Frequency of Latent HIV-1 in Resting CD4+ T Cells Using a Limiting Dilution Coculture Assay. HIV Protoc. 2016; 239–253.

6. Hill AL, Rosenbloom DIS, Fu F, Nowak MA, Siliciano RF. Predicting the outcomes of treatment to eradicate the latent reservoir for HIV-1. Proc Natl Acad Sci. 2014;111: 13475–13480. doi:10.1073/pnas.1406663111
