# Supplementary material for: HIV-1 persistence following extremely early initiation of antiretroviral therapy (ART) during acute HIV-1 infection: An observational study
Source: PLoS Med. 2017 Nov 7;14(11):e1002417. doi: 10.1371/journal.pmed.1002417 (PMC5675377; doi:10.1371/journal.pmed.1002417)
Supplement: S1 Protocol — (PDF) [file pmed.1002417.s006.pdf]

Clinical Research Protocol

ANALYTIC TREATMENT INTERRUPTION TO STUDY VIRAL RESERVOIRS AND AS A TEST FOR CURE IN HIV-INFECTED ADULTS ON LONG-TERM SUPPRESSIVE ANTIRETROVIRAL THERAPY

Protocol Number:	NA
Version Date:	Version 1
Investigational Product:	NA
IND Number:	NA
Development Phase:	NA
Sponsor:	Steven G. Deeks<br>995 Potrero Avenue, San Francisco CA, 94110<br>Steven.Deeks@ucsf.edu
Funding Organization:	amfAR
Principal Investigator:	Steven G. Deeks
Medical Monitor:	NA
Coordinating Center:	NA

Approval:

PI or Sponsor Signature (Name and Title) Date

This confidential information about an investigational product is provided for the exclusive use of investigators of this product and is subject to recall at any time. The information in this document may not be disclosed unless federal or state law or regulations require such disclosure. Subject to the foregoing, this information may be disclosed only to those persons involved in the study who have a need to know, with the obligation not to further disseminate this information.

Protocol Template © CFFT TDN Effective: 12 SEP 2007

PROTOCOL AGREEMENT

I have read the protocol specified below. In my formal capacity as Investigator, my duties include ensuring the safety of the study subjects enrolled under my supervision and providing amfAR with complete and timely information, as outlined in the protocol. It is understood that all information pertaining to the study will be held strictly confidential and that this confidentiality requirement applies to all study staff at this site. Furthermore, on behalf of the study staff and myself, I agree to maintain the procedures required to carry out the study in accordance with accepted GCP principles and to abide by the terms of this protocol.

Protocol Title: Analytic Treatment Interruption to Study Viral Reservoirs and as a Test for Cure in HIV-infected Adults on Long-term Suppressive Antiretroviral Therapy

Protocol Date: January, 2016

____ __________________

Investigator Signature Date

Steven G. Deeks, Professor of Medicine

Protocol Template © CFFT TDN Effective: 12 SEP 2007

TABLE OF CONTENTS

1 BACKGROUND 4
1.1 Overview of Non-Clinical Studies 9
1.2 Overview of Clinical Studies 9
2 STUDY RATIONALE 9
2.1 Risk / Benefit Assessment 11
3 STUDY OBJECTIVES 11
3.1 Primary Objective 12
3.2 Secondary Objectives 12
4 STUDY DESIGN 12
4.1 Study Overview 12
5 CRITERIA FOR EVALUATION 12
5.1 Primary Efficacy Endpoint 12
5.2 Secondary Efficacy Endpoints 12
5.3 Safety Evaluations 13
5.4 Other Evaluations (include only if applicable) **Error! Bookmark not defined.**
6 SUBJECT SELECTION 13
6.1 Study Population 13
6.2 Inclusion Criteria 13
6.3 Exclusion Criteria 13
7 CONCURRENT MEDICATIONS 14
7.1 Allowed **Error! Bookmark not defined.**
7.2 Prohibited **Error! Bookmark not defined.**
8 STUDY TREATMENTS 14
8.1 Method of Assigning Subjects to Treatment Groups **Error! Bookmark not defined.**
8.2 Blinding **Error! Bookmark not defined.**
8.3 Test and Control Formulation **Error! Bookmark not defined.**
8.4 Supply of Study Medication at the Site **Error! Bookmark not defined.**
8.5 Study Medication Accountability **Error! Bookmark not defined.**
8.6 Measures of Treatment Compliance **Error! Bookmark not defined.**
9 STUDY PROCEDURES AND GUIDELINES 14
9.1 Clinical Assessments 15

Protocol Template © CFFT TDN Effective: 12 SEP 2007

9.2 Clinical Laboratory Measurements (include sections as appropriate) 16
9.3 Pharmacokinetic Measurements **Error! Bookmark not defined.**
9.4 Research Laboratory Measurements (include sections as appropriate) 16
10 EVALUATIONS BY VISIT 16
10.1 Visit 1 (Day/Week/Month #) **Error! Bookmark not defined.**
10.2 Visit 2 (Day/Week/Month # include visit window) **Error! Bookmark not defined.**
10.3 Visit 3 (Day/Week/Month # include visit window) **Error! Bookmark not defined.**
10.4 Visit 4 (Day/Week/Month # include visit window) **Error! Bookmark not defined.**
10.5 Visit 5 (Follow-up or Day/Week/Month # include visit window) **Error! Bookmark not defined.**
10.6 Early Withdrawal Visit **Error! Bookmark not defined.**
11 ADVERSE EXPERIENCE REPORTING AND DOCUMENTATION 18
11.1 Adverse Events 18
11.2 Serious Adverse Experiences (SAE).. 20
11.3 Protocol Defined Important Medical Findings Requiring Real Time Reporting 20
11.4 Medical Monitoring 21
11.5 Safety Management Plan.. 4
12 DISCONTINUATION AND REPLACEMENT OF SUBJECTS 21
12.1 Withdrawal of Subjects 21
12.3 Replacement of Subjects 21
13 PROTOCOL VIOLATIONS 22
14 DATA SAFETY MONITORING (OPTIONAL SECTION – INCLUDE WHEN APPROPRIATE) ERROR! BOOKMARK NOT DEFINED.
15 STATISTICAL METHODS AND CONSIDERATIONS 22
15.1 Data Sets Analyzed 22
15.2 Demographic and Baseline Characteristics **Error! Bookmark not defined.**
15.3 Analysis of Primary Endpoint 22
15.4 Analysis of Secondary Endpoints **Error! Bookmark not defined.**
15.5 Interim Analysis **Error! Bookmark not defined.**
15.6 Sample Size and Randomization.. 23
16 DATA COLLECTION, RETENTION AND MONITORING 23
16.1 Data Collection Instruments... 23
16.2 Data Management Procedures.. 23

Protocol Template © CFFT TDN Effective: 12 SEP 2007

16.3 Data Quality Control and Reporting 23
16.4 Archival of Data 24
16.5 Availability and Retention of Investigational Records 24
16.6 Monitoring 24
16.7 Subject Confidentiality 24
17 ADMINISTRATIVE, ETHICAL, REGULATORY CONSIDERATIONS 25
17.1 Protocol Amendments 25
17.2 Institutional Review Boards and Independent Ethics Committees 25
17.3 Informed Consent Form 26
17.4 Publications 26
17.5 Investigator Responsibilities 26

Protocol Template © CFFT TDN Effective: 12 SEP 2007

LIST OF ABBREVIATIONS

Add all other abbreviations referenced in the protocol and delete any not referenced in the protocol.

**AE**	adverse event
**ALT**	alanine aminotransferase
**AST**	aspartate aminotransferase
**BUN**	blood urea nitrogen
**ART**	antiretroviral therapy
**ATI**	analytic treatment interruption
**CRF**	case report form
**CRP**	C-reactive protein
**DMC**	Data Monitoring Committee
**DSMB**	Data Safety Monitoring Board
**FDA**	Food and Drug Administration
**GCP**	Good Clinical Practice
**GGT**	gamma-glutamyl transferase
**HIPAA**	Health Insurance Portability and Accountability Act of 1996
**ICH**	International Conference on Harmonisation
**IRB**	Institutional Review Board
**IV**	intravenous
**PI**	Principal Investigator
**PK**	pharmacokinetic
**SAE**	serious adverse experience
**SGOT**	serum glutamic oxaloacetic transaminase
**SGPT**	serum glutamate pyruvate transaminase

Protocol Template © CFFT TDN Effective: 12 SEP 2007

PROTOCOL SYNOPSIS

**TITLE**	Analytic Treatment Interruption to Study Viral Reservoirs and as a Test for Cure in HIV-infected Adults on Long-term Suppressive Antiretroviral Therapy
**SPONSOR**	UCSF
**FUNDING ORGANIZATION**	amfAR
**NUMBER OF SITES**	One
**RATIONALE**	This study will enroll 20 patients who are HIV-infected with plasma HIV-1 RNA levels below the level of conventional detection (<40-75 copies/mL) for at least 12 months and CD4+ T-cell counts >350 cells/uL who will undergo a closely monitored analytic treatment interruption (ATI). Individuals who meet specific inclusion/exclusion criteria will undergo discontinuation of all antiretroviral therapy (ART). Plasma HIV RNA levels will be monitored twice weekly for 12 weeks and then weekly. ART will be resumed immediately sholud plasma HIV RNA levels increase to above 1000 copies RNA/mL. The primary efficacy outcome will be time to confirmed virologic rebound above this level.
**STUDY DESIGN**	This is a single arm uncontrolled prospective study.
**PRIMARY OBJECTIVE**	To determine the feasibility and safety of a closely monitored treatment interruption in HIV-infected adults on effective antiretroviral therapy (ART).
**SECONDARY OBJECTIVES**	To determine if treatment of acute HIV infection is associated with sustained periods of aviremia in absence of ART.<br>To determine if measures of HIV persistence or immune function predict time to rebound in individuals interrupting ART.
**NUMBER OF SUBJECTS**	20
**SUBJECT SELECTION CRITERIA**	Inclusion Criteria:<br>1 Willing and able to provide written informed consent, and<br>2 Male or female, age >/= 18 years, and<br>3 Documented HIV infection, and<br>4 Stable antiretroviral therapy for at least 12 months, and<br>5 Screening plasma HIV RNA levels below level of detection (< 40-75 copies/mL), and all available determinations in past 12 months also below level of detection (isolated single values > 75 but < 100 copies/mL will be allowed if they were preceded

Protocol Template © CFFT TDN Effective: 12 SEP 2007

	and followed by undetectable viral load determinations), and<br>6 Screening CD4+ T-cell count >350 cells/uL, and<br>7 Males must agree to use a double-barrier method of contraception throughout the study period.<br><br><u>Exclusion Criteria</u>:<br>1 Know cardiovascular or cerebrovascular disease<br>2 Known malignancy<br>3 Known severe kidney disease (CrCl < 60 mL/min via Cockroft-Gault method)<br>4 Known severe hepatic impairment (Child-Pugh Class C)<br>5 Unstable liver disease (as defined by the presence of ascites, encephalopathy, coagulopathy, hypoalbuminemia, esophageal or gastric varices, or persistent jaundice), known biliary abnormalities (with the exception of Gilbert's syndrome or asymptomatic gallstones)<br>6 Screening absolute neutrophil count <1,000 cells/mm3, platelet count < 70,000 cells/mm3, hemoglobin <8 mg/dL, aspartate aminotransferase >100 units/L, alanine aminotransferase >100 units/L<br>7 Serious illness requiring systemic treatment and/or hospitalization in preceding 6 months prior to study enrollment<br>8 Concurrent treatment with immunomodulatory drugs, and/or exposure to any immunomodulatory drug in the preceding 4 weeks prior to study enrollment (e.g. corticosteroid therapy equal to or exceeding a dose of 15mg/day of prednisone for more than 10 days, IL-2, interferon-alpha, methotrexate, cancer chemotherapy). NOTE: use of inhaled or nasal steroid is not exclusionary.<br>9 Serious medical or psychiatric illness that, in the opinion of the site investigator, would interfere with the ability to adhere to study requirements or to give informed consent.<br>10 Active drug or alcohol use or dependence that, in the opinion of the Principal Investigator, would interfere with adherence to study requirements or to give informed consent.<br>11 Unable or unwilling to use barrier protection with sexual partners
**TEST PRODUCT, DOSE, AND ROUTE OF**	NA

Protocol Template © CFFT TDN Effective: 12 SEP 2007

**ADMINISTRATION**	
**CONTROL PRODUCT, DOSE AND ROUTE OF ADMINISTRATION**	NA
**DURATION OF SUBJECT PARTICIPATION AND DURATION OF STUDY**	Subjects will be on study for up to 48 weeks.
**CONCOMMITANT MEDICATIONS**	Prohibited: Non-nucleoside reverse transcriptase inhibitors
**EFFICACY EVALUATIONS**	Plasma HIV RNA levels
**PRIMARY ENDPOINT**	Plasma HIV RNA level > 1000 copies RNA/mL
**SECONDARY ENDPOINTS**	CD4+ T cell counts
**OTHER EVALUATIONS**	Cell-associated RNA, DNA
**SAFETY EVALUATIONS**	Change in clinical safety labs<br>Incidence of adverse events
**PLANNED INTERIM ANALYSES**	When approximately 50% of patients have completed the study through Visit 12, an interim analysis for safety will be conducted by an independent data monitoring committee. Serious adverse events will be monitored by the committee on an ongoing basis throughout the study.

Protocol Template © CFFT TDN Effective: 12 SEP 2007

1 BACKGROUND

Although ART decreases HIV-associated mortality, it does not appear to completely restore health, for reasons that remain unclear. In addition, while prevention approaches have seen some significant successes in the past few years, the epidemic continues to grow both locally and globally. Perhaps the only way to fully address these and other limitations is to effectively eradicate HIV from infected persons. While complete eradication may never be feasible, a “functional cure” in which patients are able to maintain undetectable viral loads indefinitely in the absence of therapy may be possible. The best evidence for this is the existence of “elite controllers” and more recently individuals treated during very early HIV infection (“post-treatment controllers”). The study of individuals before and after initiation of ART during “hyperacute” HIV infection may provide direct insights into the pathophysiology of the earliest stages of acute HIV and the predictors and virologic/immunologic characteristics of a functional cure.

The long-term objectives of this proposal are to (1) define the safety of a closely monitored treatment interruption, (2) determine if treatment of hyperacute HIV and early HIV is associated with sustained periods of avemia during and interruption and (3) is to define how assays of HIV persistence predict what happens when therapy is interrupted. The assumption is that the smaller the size of the reservoir, the longer it will take before virus rebounds and the lower will be the level of rebound. The fact that virus rebound in those exceptional cases of near-eradication of the reservoir took many weeks to months1 provides some justification for this assumption.

1.1 Overview of Non-Clinical Studies

NA

1.2 Overview of Clinical Studies

<u>New initiatives are identifying individuals in hyperacute HIV infection and encouraging them to initiate ART immediately:</u> Given the potential individual and public health benefits of immediate ART during hyperacute/acute HIV infection, and given the greater availability of ART regimens with low pill burden and side effect profiles, treatment paradigms are now shifting towards treating individuals as early as possible. At the HIV/AIDS Clinic at SFGH (Ward 86) which has been known for its 30+ years of pioneering HIV care, the “RAPID” clinical program (Rapid ART Program Initiative for new Diagnoses) provides immediate, on-site ART initiation for all individuals diagnosed in acute HIV infection. Working with clinical, DPH, and research collaborators we have already identified over 40 individuals in acute HIV infection in San Francisco over the past year.

<u>Earlier initiation of ART during acute HIV infection leads to decreased on-treatment immune activation, improved CD4+ T cell count recovery, and decreased reservoir size:</u> Increasing data support that earlier initiation of ART during acute infection may be beneficial. In a recently published study by our group, we investigated whether early ART initiation is associated with lower on-therapy immune activation and lower measures of the viral reservoir. From a cohort of patients with early (<6 months) HIV infection, we identified individuals who started ART early (<6 months after infection, n=34) or later (≥2

Protocol Template © CFFT TDN Effective: 12 SEP 2007

years after infection, n=32), and maintained ≥2 years of virologic suppression. Early ART initiation predicted lower on-therapy CD4+ and CD8+ T cell activation compared to later ART initiation. Moreover, early ART initiation predicted 4.8-fold lower total HIV DNA levels compared to later ART initiation (p=0.005), and lower cell-associated RNA levels (p=0.035).

<u>Immediate initiation of ART during “hyperacute” HIV infection (Fiebig stages I-III) may protect long-lived Tcm cells from becoming infected:</u> In a recent unpublished study, Ananwonarich et al examined the effects of immediate ART initiation in Thai patients identified in the “hyperacute” stages of HIV infection (Fiebig Stages I-III. They identified 68 such individuals who then initiated ART after a median 15 days of HIV infection; 36% were in Fiebig stage I (NAT+, p24-), 10% were in Fiebig stage II (NAT+, p24+), and 53% were in Fiebig stage III (3rd gen EIA+, WB-). The median plasma HIV RNA level was 5.6 log10 copies/mL. Individuals started either a 5-drug (RGV/MVC/EFV/TDF/FTC) or 3-drug (EFV/TDF/FTC) ART regimen. At baseline, Fiebig I subjects had lower total HIV DNA levels in peripheral blood mononuclear cells (PBMCs) compared to both Fiebig II and III subjects. Moreover, 92% of Fiebig I subjects had undetectable integrated HIV DNA levels in PBMCs (compared to 29% and 53% in Fiebig II and III, respectively). Of the subset of subjects who underwent colorectal biopsy, Fiebig I subjects had lower total and integrated HIV DNA levels compared to Fiebig III subjects. Importantly, the HIV reservoir during acute infection was preferentially in short-lived T cell subsets (Ttm and Tem). ART initiation in these subjects restricted the reservoir in long-lived Tcm, and ART led to a rapid decay in integrated HIV DNA levels, similar to “elite” controllers and post-treatment controllers.

<u>Experience conducting intensive, single-center interventional clinical trials</u>: The SCOPE investigators (PIs: Steven Deeks, Peter Hunt, and Jeffrey Martin) have had extensive experience conducting single-center, pathogenesis-oriented, randomized, placebo-controlled clinical trials with outstanding rates of recruitment and retention of study subjects. Moreover, many of these completed and ongoing trials have included optional procedures (leukapheresis, gut biopsy, lymph node biopsy) and we have been extremely successful at recruiting participants for these more intensive substudies. We have an established IRB-approved leukapheresis protocol in place and have performed over 86 research leukaphereses on 71 unique HIV-infected subjects. In addition, we have an established IRB-approved gut biopsy protocol in place and have performed over 1020 research flexible sigmoidoscopies on 458 unique HIV-infected subjects. Finally, we have an established IRB-approved lymph node biopsy protocol in place and have performed over 65 research lymph node biopsies on 61 unique HIV-infected subjects.

2 STUDY RATIONALE

The optimal manner in which to interrupt ART remains unknown. Given that an interruption may prove to be the most informative method to quantify the size of the reservoir and/or to determine the efficacy of a curative intervention, a number of groups--including the AIDS Clinical Trials Group (ACTG) and NIAID scientific leadership--have developed an approach which minimizes risk to the study participant (which has been termed an "Intensely Monitored Antiretroviral Pause", or MAP). This approach is being used in prospective studies of the reservoir, including ACTG 5345 (Dr. Deeks is a vice

Protocol Template © CFFT TDN Effective: 12 SEP 2007

chair on this study). This approach is also being used in studies of hyperacute HIV infection and a variety of NIAD-supported interventional studies. Our approach outlined below is identical to that being used in these NIH-supported studies.

Our treatment interruption protocol is based in part on on previous interruption studies performed with NIH funding by Tim Schacker and colleagues. In this study, HIV-infected adults interrupted therapy and were monitored in the clinic two to three times per week. ART was initiated once viremia became detectable. Most subjects initiated therapy before viral load increased to above 10,000 copies RNA/mL. No drug resistance occurred during the interruption and there were no negative adverse events. All subjects were able to successfully resume suppressive therapy before there was any obvious decline in peripheral CD4+ T cell counts.

2.1 Risk / Benefit Assessment

We are aware that interrupting ART has potential for significant risks. We will use a conservative study design to minimize risk. All individuals will be on a regimen that includes antiretroviral drugs with rapid clearance. This will minimize the chance that drugs will persist long enough to select for drug resistance in the rebounding virus. We only include individuals with high CD4+ T cell counts to minimize the risk of developing clinical relevant immunodeficiency once the virus rebounds. We will monitored individuals twice weekly during the first 12 weeks and then weekly, and resume therapy as soon as plasma HIV RNA levels increase to above 1000 copies RNA/mL. In a recent pilot study using this approach, study participants were exposed to rebounding virus for only a few days and there were no significant adverse events.

Subjects will be counseled during the informed consent process regarding the benefits and risks of interrupting antiretroviral therapy. Study participants will be counselled on the risks of transmission of HIV should their plasma HIV-1 RNA levels increase after ART interruption. We have minimized blood draws to those necessary for the study. We will monitor side-effects through patient interviews at all study visits and routine laboratories.

Our study has been designed to minimize the time from when virus becomes detectable and when ART is resumed. All participants will be seen twice weekly for the first 12 weeks (Weeks 0-12) of the ATI. Participants will be seen weekly for the next 12 weeks of the ATI (Weeks 12-24). A plasma HIV-1 RNA level will be performed on all of these visits. In a pilot study performed at the University of Minnesota, individuals who interrupted therapy in a comparable setting were able to resume therapy before peak viremia and before any detectable changes in circulating CD4+ T cell counts were observed.

Individuals who interrupt drugs that persist for days to weeks are at the risk of developing a drug resistant variant. This risk is particuarly relevant when interrupting non-nucleoside reverse transcriptase inhibitors (NNRTIs), as these drugs often have very long half lives and as resistance to these drugs is easy to generate. We will exclude individuals who are on an NNRTI-based regimen unless they can be switched to another regimen.

The principal investigator will monitor the study. All participants will be followed for possible adverse events and unanticipated problems throughout their involvement in the study. At each visit, study personnel will elicit subject input as to discomforts or adverse

Protocol Template © CFFT TDN Effective: 12 SEP 2007

experiences while taking the medications. A complete blood count, complete metabolic panel, CD4+ T cell count, and plasma HIV-1 RNA level will be performed on most visits. The principal investigator will obtain these safety data in "real-time" (i.e., within 1-10 days of a study visit) when laboratory values become available.

We will also develop an independent Safety Monitoring Committee (SMC) prior to the initiation of the study. The SMC will be composed of 3 independent individuals from the scientific community selected by the principal investigator and co-principal investigator. The SMC will meet at 4, 8, 12, 24, and 36 weeks after the enrollment of the first subject and at 48 weeks after the enrollment of the last subject. The SMC will review study progress, efficacy data, all interim and total adverse events, and unanticipated problems involving risk to participants. Reviews will be communicated to the CHR, study sponsor, and/or federal agencies (as appropriate).

3 STUDY OBJECTIVES

3.1 Primary Objective

To determine the feasibility and safety of a closely monitored treatment interruption in HIV-infected adults on effective antiretroviral therapy (ART).

3.2 Secondary Objectives

To determine if treatment of acute HIV infection is associated with sustained periods of aviremia in absence of ART.

To determine if measures of HIV persistence or immune function predict time to rebound in individuals interrupting ART.

4 STUDY DESIGN

4.1 Study Overview

HIV-infected participants with plasma HIV-1 RNA levels below the level of conventional detection (<40-75 copies/mL) for at least 12 months and CD4+ T-cell counts >350 cells/uL will undergo a closely monitored analytic treatment interruption (ATI). Individuals who meet specific inclusion/exclusion criteria will undergo discontinuation of all antiretroviral therapy (ART). Plasma HIV RNA levels will be monitored twice weekly for 12 weeks and then weekly. ART will be resumed immediately should plasma HIV RNA levels increase to above 1000 copies RNA/mL. The primary efficacy outcome will be time to confirmed virologic rebound above this level.

5 CRITERIA FOR EVALUATION

5.1 Primary Efficacy Endpoint

- Plasma HIV RNA level > 1000 copies RNA/mL

5.2 Secondary Efficacy Endpoints

- CD4+ T cell counts

Protocol Template © CFFT TDN Effective: 12 SEP 2007

- CD4/CD8 ratio
- Adverse events

5.3 Safety Evaluations

- Change in CD4+ T cell counts
- HIV drug resistasnce (genotype)
- Incidence of adverse events

6 SUBJECT SELECTION

6.1 Study Population

Subjects with HIV infection who meet the inclusion and exclusion criteria will be eligible for participation in this study.

6.2 Inclusion Criteria

1. Willing and able to provide written informed consent, and
2. Male or female, age >/= 18 years, and
3. Documented HIV infection, and
4. Stable antiretroviral therapy for at least 12 months, and
5. Screening plasma HIV RNA levels below level of detection (< 40-75 copies/mL), and all available determinations in past 12 months also below level of detection (isolated single values > 75 but < 100 copies/mL will be allowed if they were preceded and followed by undetectable viral load determinations), and
6. Screening CD4+ T-cell count >350 cells/uL, and
7. Males must agree to use a double-barrier method of contraception throughout the study period.

6.3 Exclusion Criteria

1. Pregnant, breastfeeding, or unwilling to practice birth control during participation in the study
2. Know cardiovascular or cerebrovascular disease
3. Known malignancy
4. Known severe kidney disease (CrCl < 60 mL/min via Cockroft-Gault method)
5. Known severe hepatic impairment (Child-Pugh Class C)
6. Unstable liver disease (as defined by the presence of ascites, encephalopathy, coagulopathy, hypoalbuminemia, esophageal or gastric varices, or persistent jaundice), known biliary abnormalities (with the exception of Gilbert's syndrome or asymptomatic gallstones)

Protocol Template © CFFT TDN Effective: 12 SEP 2007

7. Screening absolute neutrophil count <1,000 cells/mm3, platelet count < 70,000 cells/mm3, hemoglobin <8 mg/dL, aspartate aminotransferase >100 units/L, alanine aminotransferase >100 units/L
8. Serious illness requiring systemic treatment and/or hospitalization in preceding 6 months prior to study enrollment
9. Concurrent treatment with immunomodulatory drugs, and/or exposure to any immunomodulatory drug in the preceding 4 weeks prior to study enrollment (e.g. corticosteroid therapy equal to or exceeding a dose of 15mg/day of prednisone for more than 10 days, IL-2, interferon-alpha, methotrexate, cancer chemotherapy). NOTE: use of inhaled or nasal steroid is not exclusionary.
10. Serious medical or psychiatric illness that, in the opinion of the site investigator, would interfere with the ability to adhere to study requirements or to give informed consent.
11. Active drug or alcohol use or dependence that, in the opinion of the Principal Investigator, would interfere with adherence to study requirements or to give informed consent.
12. Unable or unwilling to use barrier protection with sexual partners.

7 CONCURRENT MEDICATIONS

All subjects should be maintained on the same medications throughout the entire study period, as medically feasible, with no introduction of new chronic therapies. Subjects on a non-nucleoside reverse transcriptaste inhibitor will not be enrolled into this study.

8 STUDY TREATMENTS

No treatments will be provided by the study.

9 STUDY PROCEDURES AND GUIDELINES

Prior to conducting any study-related activities, written informed consent and the Health Insurance Portability and Accountability Act (HIPAA) authorization must be signed and dated by the subject. If appropriate, assent must also be obtained prior to conducting any study-related activities.

The proposed research plan is made possible by the UCSF SCOPE cohort an established prospective, clinic-based cohort study of HIV-infected adults. To date, SCOPE has enrolled over 2000 participants, including over 800 participants who have maintained virologic suppression on ART. Detailed interviews are conducted every 4 months, including questions regarding medications, medication adherence, and intercurrent illnesses. Plasma HIV-1 RNA levels and CD4+ T cell counts are measured, and PBMCs and plasma samples are obtained at each visit. The SCOPE study has a long track record of supporting investigator-initiated studies. Protocols are already in place for specimen collection and archiving, medication storage, safety monitoring, and data verification and management. The SCOPE team has also had extensive experience with all regulatory aspects of conducting single-center interventional clinical trials such as that outlined here.

Protocol Template © CFFT TDN Effective: 12 SEP 2007

9.1 Clinical Assessments

9.1.1 Concomitant Medications

All concomitant medication and concurrent therapies will be documented screening and baseline visits.

9.1.2 Demographics

Demographic information (date of birth, gender, race) will be recorded at Screening.

9.1.3 Medical History

Relevant medical history, including history of current disease, other pertinent respiratory history, and information regarding underlying diseases will be recorded at Screening.

9.1.4 Physical Examination

A general medical evaluation (including determination of vital signs) will be performed at the screening visit and at Weeks 0, 1, 2, 4, 8, 12, 16, 20, and 24.

9.1.5 Other Clinical Procedures

Blood collection: Blood collection will occur at all visits and will be timed to stay within Red Cross Guidelines (less than 500 mL every 8 weeks).

Leukapheresis: Our group already has an UCSF CHR-approved study in place (PI: Steven Deeks, MD, "The Use of Leukapheresis to Support HIV Pathogenesis Studies", 10-03244) which will allow for leukapheresis to be performed on HIV-infected subjects. Leukapheresis is a procedure that has been used in clinical practice for over 25 years, in which granulocytes (and in some cases lymphocytes) are selectively harvested but red cells and other blood components are returned to the patient.

Colorectal biopsy: Our group already has an UCSF CHR-approved study in place (PI: Peter Hunt, MD, "Impact of HIV on Gut-Associated Lymphoid Tissue [GALT]," 10-01218) which will allow for specimen collection and archiving of GALT samples. Participants in this study will have the option to participate (co-enroll) in the colorectal biopsy study at least 12 weeks prior to Day 0 and within 12 weeks after meeting criteria to restart ART.

Inguinal LN biopsy: Our group already has an UCSF CHR-approved study in place (PI: Steven Deeks, MD, "The use of lymph node biopsies to support HIV pathogenesis studies," 10-03606) which will allow for specimen collection and archiving of inguinal lymph node samples. Participants in this study will have the option to participate (co-enroll) in the colorectal biopsy study at least 12 weeks prior to Day 0 and within 12 weeks after meeting criteria to restart ART.

9.1.6 Adverse Events

Information regarding occurrence of adverse events will be captured throughout the study.

Protocol Template © CFFT TDN Effective: 12 SEP 2007

9.2 Clinical Laboratory Measurements

9.2.1 Hematology

Blood will be obtained and sent to each site's clinical hematology lab for a complete blood count (hemoglobin, hematocrit, red blood cell count, white blood cell count, white blood cell differential, and platelet count).

9.2.2 Blood Chemistry Profile

Blood will be obtained and sent to each site's clinical chemistry lab for determination of serum sodium, potassium, chloride, bicarbonate, random glucose, BUN, creatinine, aspartate aminotransferase (AST/SGOT), alanine aminotransferase (ALT/SGPT), alkaline phosphatase, total bilirubin, direct bilirubin, gamma-glutamyl transferase (GGT), albumin and LDH.

9.2.3 Pregnancy Test

A serum pregnancy test will be obtained from female subjects who are of childbearing age prior to their participation in the study.

9.3 Research Laboratory Measurements

<u>Detection of cell-associated RNA (PBMCs):</u> Using similar approaches outlined in our multiple publications, cell-associated HIV RNA will be measured from 5x106 PBMCs.

<u>Detection of total proviral HIV DNA (PBMCs):</u> Using similar approaches outlined in our multiple publications, we will measure total proviral HIV DNA.

<u>Measurement of immune activation and HIV-specific responses (PBMCs):</u> Our group has had extensive experience measuring immune activation in the context of observational and interventional studies. Among CD4+ and CD8+ T cells, we will examine the proportion of cells that are expressing CD38 (a multifunctional ectoenzyme expressed by activated T cells). The activation marker CD38 has been found to be an important predictor of disease outcome among HIV infected persons. We will also examine the concurrent expression of HLA-DR as resting naïve T cells also may express CD38 in the absence of activation; gating on cells concurrently expressing HLA-DR excludes these resting naïve T cells. We will measure the proportion of activated (CD38+/HLA-DR+) CD4+ and CD8+ T-cells. In addition, we will measure by cytokine flow cytometry the proportion of CD4+ and CD8+ T-cells co-expressing IFNgand IL2 to HIV Gag-peptides. We will also measure T-cell exhaustion markers (PD-1, Tim-3, and Lag-3) as these markers (measured prior to ART) have been shown to be strongly predictive of time to viremia during treatment interruption.

10 EVALUATIONS BY VISIT

Participants will be consented by the Principal Investigator or the research team before any procedures take place. All efforts will be made to have all visits occur between 8am and 11am, in order to account for diurnal variation in CD4+ T cell count and to allow for same-day shipping and storage of samples at the UCSF AIDS Specimen Bank.

Protocol Template © CFFT TDN Effective: 12 SEP 2007

Unique Identifier: Once a participant is identified as potentially eligible by phone screening and is to be scheduled for a screening visit, the next available unique identifier (SCOPE ID) will be assigned. Once a SCOPE ID is assigned, it cannot be reassigned to another subject.

- Week -4: All screening, enrollment, and consent procedures will be completed, including screening laboratory and clinical evaluations.
- Week -2: Individuals taking an ART regimen that contains a non-nucleoside reverse transcriptase inhibitor (NNRTI) will be switched to a regimen that substitutes the integrase inhibitor dolutegravir for the NNRTI for two weeks prior to ATI. This will minimize the risk of HIV resistance developing to the NNRTI. Individuals on other ART regimens that do not contain an NNRTI drug will continue their usual ART medications until ATI at Week 0.
- Week 0: All antiretroviral medicines will be stopped.
- Weeks 1-12: Twice weekly clinical and laboratory monitoring will occur. Any participant who meets the virologic criteria for treatment re-initiation will be advised to restart ART using the same regimen they were taking before study enrollment. The study visit prior to reinitiating ART will be follow the data and specimen collection schedule for week 24.
- Weeks 12-24: Once weekly clinical and laboratory monitoring will occur. Any participant who meets the virologic criteria for treatment re-initiation will be advised to restart ART using the same regimen they were taking before study enrollment. The study visit prior to reinitiating ART will be follow the data and specimen collection schedule for week 24.
- Week 24: Individuals who remain off ART and have not yet reached any of the criteria to reinitiate ART will discuss with the Principal Investigator the option of reinitiating ART at this time.
- Post-week 24: After completing all study visits and procedures, participants will continue follow-up under SCOPE protocol.

Monitoring during ATI: Plasma HIV-1 RNA levels will be measured at Week 0. Monitoring of plasma HIV-1 RNA levels during ATI will vary over the course of the ATI:

- Weeks 1-12: Twice weekly.
- Weeks 12-24: Once weekly.

If plasma HIV-1 RNA levels becomes detectable (>40-75 copies/mL), then testing will be repeated every 3 days until plasma HIV-1 RNA levels <40-75 copies/mL or the participant meets criteria for re-initiation of ART.

Reinitiation of ART: Specific criteria for reinitiation of ART after ATI are:

- Plasma HIV-1 RNA >1,000 copies/mL on 2 consecutive determinations, OR
- A single plasma HIV-1 RNA >10,000 copies/mL, OR
- Pregnancy, OR

Protocol Template © CFFT TDN Effective: 12 SEP 2007

- Subject requests re-initiation of ART

Monitoring after restarting ART: Any participant who meets the virologic criteria for treatment re-initiation will be advised to restart ART using the same regimen they were taking before study enrollment. If ART is restarted due to rebound viremia with HIV-1 RNA > 1,000 copies/mL, then HIV genotype testing will be done at time of reinitiating ART. The results of genotype testing will be made available to guide any necessary ART modification. The final choice of regimen will be determined by discussion between the participant and the Principal Investigator. Once ART has been reinitiated, plasma HIV-1 RNA levels will be checked weekly until undetectable (<40-75 copies/mL) and then every other week until 2 consecutive measurements are undetectable.

Leukapheresis: Our group already has an UCSF CHR-approved study in place (PI: Steven Deeks, MD, "The Use of Leukapheresis to Support HIV Pathogenesis Studies", 10-03244) which will allow for leukapheresis to be performed on HIV-infected subjects. Leukapheresis is a procedure that has been used in clinical practice for over 25 years, in which granulocytes (and in some cases lymphocytes) are selectively harvested but red cells and other blood components are returned to the patient.

Colorectal biopsy: Our group already has an UCSF CHR-approved study in place (PI: Peter Hunt, MD, "Impact of HIV on Gut-Associated Lymphoid Tissue [GALT]," 10-01218) which will allow for specimen collection and archiving of GALT samples. Participants in this study will have the option to participate (co-enroll) in the colorectal biopsy study at least 12 weeks prior to Day 0 and within 12 weeks after meeting criteria to restart ART.

Inguinal LN biopsy: Our group already has an UCSF CHR-approved study in place (PI: Steven Deeks, MD, "The use of lymph node biopsies to support HIV pathogenesis studies," 10-03606) which will allow for specimen collection and archiving of inguinal lymph node samples. Participants in this study will have the option to participate (co-enroll) in the colorectal biopsy study at least 12 weeks prior to Day 0 and within 12 weeks after meeting criteria to restart ART.

11 EXPERIENCE REPORTING AND DOCUMENTATION

11.1 Adverse Events

An adverse event (AE) is any untoward medical occurrence in a clinical investigation of a patient administered a pharmaceutical product and that does not necessarily have a causal relationship with the treatment. An AE is therefore any unfavorable and unintended sign (including an abnormal laboratory finding), symptom or disease temporally associated with the administration of an investigational product, whether or not related to that investigational product. An unexpected AE is one of a type not identified in nature, severity, or frequency in the current Investigator's Brochure or of greater severity or frequency than expected based on the information in the Investigator's Brochure.

The Investigator will probe, via discussion with the subject, for the occurrence of AEs during each subject visit and record the information in the site's source documents. Adverse events will be recorded in the patient CRF. Adverse events will be described by

Protocol Template © CFFT TDN Effective: 12 SEP 2007

duration (start and stop dates and times), severity, outcome, treatment and relation to study drug, or if unrelated, the cause.

AE Severity

The National Cancer Institute's Common Terminology Criteria for Adverse Events (CTCAE) Version 3.0 should be used to assess and grade AE severity, including laboratory abnormalities judged to be clinically significant. The modified criteria can be found in the study manual. If the experience is not covered in the modified criteria, the guidelines shown in Table 1 below should be used to grade severity. It should be pointed out that the term "severe" is a measure of intensity and that a severe AE is not necessarily serious.

Table 1. AE Severity Grading

Severity (Toxicity Grade)	Description
Mild (1)	Transient or mild discomfort; no limitation in activity; no medical intervention or therapy required. The subject may be aware of the sign or symptom but tolerates it reasonably well.
Moderate (2)	Mild to moderate limitation in activity, no or minimal medical intervention/therapy required.
Severe (3)	Marked limitation in activity, medical intervention/therapy required, hospitalizations possible.
Life-threatening (4)	The subject is at risk of death due to the adverse experience as it occurred. This does not refer to an experience that hypothetically might have caused death if it were more severe.

AE Relationship to Study Drug

The relationship of an AE to the study drug should be assessed using the following the guidelines in Table 2.

Table 2. AE Relationship to Study Drug

Relationship to Drug	Comment
Definitely	Previously known toxicity of agent; or an event that follows a reasonable temporal sequence from administration of the drug; that follows a known or expected response pattern to the suspected drug; that is confirmed by stopping or reducing the dosage of the drug; and that is not explained by any other reasonable hypothesis.
Probably	An event that follows a reasonable temporal sequence from administration of the drug; that follows a known or expected response pattern to the suspected drug; that is confirmed by stopping or reducing the dosage of the drug; and that is unlikely to be explained by the known characteristics of the subject's clinical state or by other interventions.
Possibly	An event that follows a reasonable temporal sequence from administration of the drug; that follows a known or expected response pattern to that suspected drug; but that could readily have been produced by a number of other factors.

Protocol Template © CFFT TDN Effective: 12 SEP 2007

Unrelated	An event that can be determined with certainty to have no relationship to the study drug.

11.2 Serious Adverse Experiences (SAE)

An SAE is defined as any AE occurring at any dose that results in any of the following outcomes:

- death
- a life-threatening adverse experience
- inpatient hospitalization or prolongation of existing hospitalization
- a persistent or significant disability/incapacity
- a congenital anomaly/birth defect

Other important medical events may also be considered an SAE when, based on appropriate medical judgment, they jeopardize the subject or require intervention to prevent one of the outcomes listed.

11.2.1 Serious Adverse Experience Reporting

Study sites will document all SAEs that occur (whether or not related to study drug) per UCSF CHR Guidelines. The collection period for all SAEs will begin after informed consent is obtained and end after procedures for the final study visit have been completed.

In accordance with the standard operating procedures and policies of the local Institutional Review Board (IRB)/Independent Ethics Committee (IEC), the site investigator will report SAEs to the IRB/IEC.

11.3 Protocol Defined Important Medical Findings Requiring Real Time Reporting

Blood drawing (venipuncture) risks. Drawing blood from a vein may cause some discomfort, bleeding, or bruising where the needle enters the skin, and rarely, fainting or infection may occur. Up to a total of 1500 mL (about 3 pints) of blood will be drawn over the entire study period. No more than 480 mL (2 cups) of blood will be drawn over any two-month period. This is within Red Cross Guidelines (less than 500 mL every two months). Risks of blood collection include anemia (low blood counts). Symptoms of anemia include tiredness, weakness and dizziness. Subjects will be checked for anemia at each visit. If the investigator feels that a subject is at significant risk for anemia, the amount of blood collected will be reduced. If hemoglobin falls below 9 g/dL or hematocrit falls below 27%, subjects will have 5 mL (1 teaspoon) of blood drawn to check hemoglobin and hematocrit. Other than the blood required to check hemoglobin and hematocrit, subjects will not have more blood drawn until hemoglobin rises above 9 g/dL or hematocrit rises above 27%.

Acute Retroviral Syndrome (ARS). Participants who develop acute retroviral syndrome will re-start ART as soon as possible (ideally, within 1 to 3 days). Signs and symptoms of acute retroviral syndrome include fever, headache, malaise, swollen lymph nodes, and joint or other body aches.

Protocol Template © CFFT TDN Effective: 12 SEP 2007

Drug resistance. Individuals who interrupt drugs that persist for days to weeks are at the risk of developing a drug resistant variant. This risk is particularly relevant when interrupting non-nucleoside reverse transcriptase inhibitors (NNRTIs), as these drugs often have very long half-lives and as resistance to these drugs is easy to generate. We will exclude individuals who are on an NNRTI-based regimen unless they can be switched to another regimen.

11.4 Medical Monitoring

NA

12 DISCONTINUATION AND REPLACEMENT OF SUBJECTS

12.1 Early Discontinuation

A subject may be discontinued from study treatment at any time if the subject, the investigator, or the Sponsor feels that it is not in the subject’s best interest to continue. The following is a list of possible reasons for study treatment discontinuation:

- Subject withdrawal of consent (or assent)
- Subject is not compliant with study procedures
- Adverse event that in the opinion of the investigator would be in the best interest of the subject to discontinue study treatment
- Protocol violation requiring discontinuation of study treatment
- Lost to follow-up
- Sponsor request for early termination of study
- Positive pregnancy test (females)

If a subject is withdrawn from treatment due to an adverse event, the subject will be followed and treated by the Investigator until the abnormal parameter or symptom has resolved or stabilized.

All subjects who discontinue study treatment should come in for an early discontinuation visit as soon as possible and then should be encouraged to complete all remaining scheduled visits and procedures.

All subjects are free to withdraw from participation at any time, for any reason, specified or unspecified, and without prejudice.

Reasonable attempts will be made by the investigator to provide a reason for subject withdrawals. The reason for the subject’s withdrawal from the study will be specified in the subject’s source documents.

12.3 Withdrawal of Subjects from the Study

A subject may be withdrawn from the study at any time if the subject, the investigator, or the Sponsor feels that it is not in the subject’s best interest to continue.

Protocol Template © CFFT TDN Effective: 12 SEP 2007

All subjects are free to withdraw from participation at any time, for any reason, specified or unspecified, and without prejudice.

Reasonable attempts will be made by the investigator to provide a reason for subject withdrawals. The reason for the subject's withdrawal from the study will be specified in the subject's source documents. As noted above, subjects who discontinue study treatment early should have an early discontinuation visit.

12.4 Replacement of Subjects

Subjects who withdraw from the study will not be replaced.

13 PROTOCOL VIOLATIONS

A protocol violation occurs when the subject or investigator, fails to adhere to significant protocol requirements affecting the inclusion, exclusion, subject safety and primary endpoint criteria. Protocol violations for this study include, but are not limited to, the following:

- Failure to meet inclusion/exclusion criteria
- Use of a prohibited concomitant medication
- Failure to comply with Good Clinical Practice (GCP) guidelines will also result in a protocol violation.

When a protocol violation occurs, it will be discussed with the investigator and a Protocol Violation Form detailing the violation will be generated. This form will be signed by a Sponsor representative and the Investigator. A copy of the form will be filed in the site's regulatory binder and in the Sponsor's files.

14 STATISTICAL METHODS AND CONSIDERATIONS

Prior to the analysis of the final study data, a detailed Statistical Analysis Plan (SAP) will be written describing all analyses that will be performed. The SAP will contain any modifications to the analysis plan described below.

14.1 Data Sets Analyzed

All eligible patients who are enrolled into the study and interrupt therapy will be included in the safety analysis.

14.2 Data Analysis Plan

We will follow standard good statistical practices, including examination of summary statistics, assessment of model assumptions, examination and presentation of graphical depictions of the data, assessing the impact of influential data points, and interpretation that reflects the quantitative information provided by estimated effects and their confidence intervals, rather than an exclusive focus on whether or not $p<0.05$.

Some measures, such as integrated DNA and cell-associated RNA, will likely be better modeled by negative binomial regression than by simpler methods such as linear regression or t-tests, because they tend to have a skewed distribution, cannot be negative,

Protocol Template © CFFT TDN Effective: 12 SEP 2007

may equal zero (precluding logarithmic transformation), and are more variable when values are higher. In addition, it may be desirable to account for the amount of input to the assays, which is readily done in negative binomial models by inclusion of an “exposure” variable. In these cases, and also when linear regression or t-tests of logarithmically transformed measurements are appropriate, we will report relative effects (percentage or fold). Because nonparametric methods such as the Wilcoxon signed-rank test produce only p-values with no quantitative effect estimates, we will use these only as confirmatory analyses or when no quantitative analysis appears viable.

14.3 Sample Size and Randomization

Because this is a pilot study, the sample size will be limited to a number that can be feasibly enrolled in a 12 month period. These data may contribute to future funding of similar, larger studies. Only individuals who have a relatively low viral reservoir will undergo analytic treatment interruption. At this time, it is not expected that there will be more than 20 such individuals who meet this criteria

15 DATA COLLECTION, RETENTION AND MONITORING

15.1 Data Collection Instruments

The Investigator will prepare and maintain adequate and accurate source documents designed to record all observations and other pertinent data for each subject treated with the study drug.

Detailed interviews (as part of the existing SCOPE protocol) will be conducted at each visit, including questions regarding current medications, medication adherence, intercurrent illnesses, and hospitalizations. We will use the UCSF CHR-approved SCOPE questionnaires for this study (PI, Steve Deeks, MD, “Study on Consequences of Protease Inhibitor Era [SCOPE]: A Prospective Study” 10-01330).

Subjects will not be identified by name in the study database or on any study documents to be collected by the Sponsor (or designee), but will be identified by a four digit number.

The Investigator is responsible for all information collected on subjects enrolled in this study. All data collected during the course of this study must be reviewed and verified for completeness and accuracy by the Investigator.

15.2 Data Management Procedures

The data will be entered into a validated database. The Data Management group will be responsible for data processing, in accordance with procedural documentation. Database lock will occur once quality assurance procedures have been completed.

All procedures for the handling and analysis of data will be conducted using good computing practices meeting FDA guidelines for the handling and analysis of data for clinical trials.

15.3 Data Quality Control and Reporting

After data have been entered into the study database, a system of computerized data validation checks will be implemented and applied to the database on a regular basis.

Protocol Template © CFFT TDN Effective: 12 SEP 2007

All data will be managed by the UCSF Data Coordinating Center in the Department of Epidemiology and Biostatistics. Issues related to data management, specimen storage, and data analysis will be directed by Dr. Jeffrey Martin, who co-directs the SCOPE cohort with Dr. Deeks. Study participants will complete an interviewer-administered questionnaire modified to support the unique aspects of our proposed study (including collection of detailed information regarding sexual activities in both the study participant, and his or her partners). The standard system is compliant with all Federal Government confidentiality guidelines.

15.4 Archival of Data

The database is safeguarded against unauthorized access by established security procedures; appropriate backup copies of the database and related software files will be maintained. Databases are backed up by the database administrator in conjunction with any updates or changes to the database.

At critical junctures of the protocol (e.g., production of interim reports and final reports), data for analysis is locked and cleaned per established procedures.

15.5 Availability and Retention of Investigational Records

The Investigator must make study data accessible to the monitor, other authorized representatives of the Sponsor (or designee), IRB/IEC, and Regulatory Agency (e.g., FDA) inspectors upon request. A file for each subject must be maintained that includes the signed Informed Consent, HIPAA Authorization and Assent Form and copies of all source documentation related to that subject. The Investigator must ensure the reliability and availability of source documents from which the information on the CRF was derived.

All study documents (patient files, signed informed consent forms, copies of CRFs, Study File Notebook, etc.) must be kept secured for a period of two years following marketing of the investigational product or for two years after centers have been notified that the IND has been discontinued. There may be other circumstances for which the Sponsor is required to maintain study records and, therefore, the Sponsor should be contacted prior to removing study records for any reason.

15.6 Monitoring

Monitoring visits will be conducted by representatives of the Sponsor according to the U.S. CFR Title 21 Parts 50, 56, and 312 and ICH Guidelines for GCP (E6). By signing this protocol, the Investigator grants permission to the Sponsor (or designee), and appropriate regulatory authorities to conduct on-site monitoring and/or auditing of all appropriate study documentation.

15.7 Subject Confidentiality

In order to maintain subject confidentiality, only a four digit subject number will identify all study subjects on CRFs and other documentation submitted to the Sponsor. Additional subject confidentiality issues (if applicable) are covered in the Clinical Study Agreement.

Protocol Template © CFFT TDN Effective: 12 SEP 2007

16 ADMINISTRATIVE, ETHICAL, REGULATORY CONSIDERATIONS

The study will be conducted according to the Declaration of Helsinki, Protection of Human Volunteers (21 CFR 50), Institutional Review Boards (21 CFR 56), and Obligations of Clinical Investigators (21 CFR 312).

To maintain confidentiality, all laboratory specimens, evaluation forms, reports and other records will be identified by a coded number and initials only. All study records will be kept in a locked file cabinet and code sheets linking a patient's name to a patient identification number will be stored separately in another locked file cabinet. Clinical information will not be released without written permission of the subject, except as necessary for monitoring by the FDA. The Investigator must also comply with all applicable privacy regulations (e.g., Health Insurance Portability and Accountability Act of 1996, EU Data Protection Directive 95/46/EC).

16.1 Protocol Amendments

Any amendment to the protocol will be written by PI. Protocol amendments cannot be implemented without prior written IRB/IEC approval except as necessary to eliminate immediate safety hazards to patients. A protocol amendment intended to eliminate an apparent immediate hazard to patients may be implemented immediately, provided the IRBs are notified within five working days.

16.2 Institutional Review Boards and Independent Ethics Committees

The protocol and consent form will be reviewed and approved by the IRB/IEC of each participating center prior to study initiation. Serious adverse experiences regardless of causality will be reported to the IRB/IEC in accordance with the standard operating procedures and policies of the IRB/IEC, and the Investigator will keep the IRB/IEC informed as to the progress of the study. The Investigator will obtain assurance of IRB/IEC compliance with regulations.

Any documents that the IRB/IEC may need to fulfill its responsibilities (such as protocol, protocol amendments, Investigator's Brochure, consent forms, information concerning patient recruitment, payment or compensation procedures, or other pertinent information) will be submitted to the IRB/IEC. The IRB/IECs written unconditional approval of the study protocol and the informed consent form will be in the possession of the Investigator before the study is initiated. The IRB/IECs unconditional approval statement will be transmitted by the Investigator to any sponsor prior to the shipment of study supplies to the site. This approval must refer to the study by exact protocol title and number and should identify the documents reviewed and the date of review.

Protocol and/or informed consent modifications or changes may not be initiated without prior written IRB/IEC approval except when necessary to eliminate immediate hazards to the patients or when the change(s) involves only logistical or administrative aspects of the study. Such modifications will be submitted to the IRB/IEC and written verification that the modification was submitted and subsequently approved should be obtained.

The IRB/IEC must be informed of revisions to other documents originally submitted for review; serious and/or unexpected adverse experiences occurring during the study in accordance with the standard operating procedures and policies of the IRB; new

Protocol Template © CFFT TDN Effective: 12 SEP 2007

information that may affect adversely the safety of the patients of the conduct of the study; an annual update and/or request for re-approval; and when the study has been completed.

16.3 Informed Consent Form

Informed consent will be obtained in accordance with the Declaration of Helsinki, ICH GCP, US Code of Federal Regulations for Protection of Human Subjects (21 CFR 50.25[a,b], CFR 50.27, and CFR Part 56, Subpart A), the Health Insurance Portability and Accountability Act (HIPAA, if applicable), and local regulations.

The Investigator will prepare the informed consent form, assent and HIPAA authorization and provide the documents to the Sponsor or designee for approval prior to submission to the IRB/IEC. The consent form generated by the Investigator must be acceptable to the Sponsor and be approved by the IRB/IEC. The written consent document will embody the elements of informed consent as described in the International Conference on Harmonisation and will also comply with local regulations. The Investigator will send an IRB/IEC-approved copy of the Informed Consent Form to the Sponsor (or designee) for the study file.

A properly executed, written, informed consent will be obtained from each subject prior to entering the subject into the trial. Information should be given in both oral and written form and subjects must be given ample opportunity to inquire about details of the study. If appropriate and required by the local IRB/IEC, assent from the subject will also be obtained. If a subject is unable to sign the informed consent form (ICF) and the HIPAA authorization, a legal representative may sign for the subject. A copy of the signed consent form (and assent) will be given to the subject and the original will be maintained with the subject's records.

16.4 Publications

The preparation and submittal for publication of manuscripts containing the study results shall be in accordance with a process determined by mutual written agreement among the study Sponsor and participating institutions. The publication or presentation of any study results shall comply with all applicable privacy laws, including, but not limited to, the Health Insurance Portability and Accountability Act of 1996.

16.5 Investigator Responsibilities

By signing the Agreement of Investigator form, the Investigator agrees to:

1. Conduct the study in accordance with the protocol and only make changes after notifying the Sponsor (or designee), except when to protect the safety, rights or welfare of subjects.
    1. Personally conduct or supervise the study (or investigation).
    2. Ensure that the requirements relating to obtaining informed consent and IRB review and approval meet federal guidelines, as stated in § 21 CFR, parts 50 and 56.
    3. Report to the Sponsor or designee any AEs that occur in the course of the study, in accordance with §21 CFR 312.64.

Protocol Template © CFFT TDN Effective: 12 SEP 2007

4. Ensure that all associates, colleagues and employees assisting in the conduct of the study are informed about their obligations in meeting the above commitments.
5. Maintain adequate and accurate records in accordance with §21 CFR 312.62 and to make those records available for inspection with the Sponsor (or designee).
6. Ensure that an IRB that complies with the requirements of §21 CFR part 56 will be responsible for initial and continuing review and approval of the clinical study.
7. Promptly report to the IRB and the Sponsor (or designee) all changes in the research activity and all unanticipated problems involving risks to subjects or others (to include amendments and IND safety reports).
8. Seek IRB approval before any changes are made in the research study, except when necessary to eliminate hazards to the patients/subjects.
9. Comply with all other requirements regarding the obligations of clinical investigators and all other pertinent requirements listed in § 21 CFR part 312.

Protocol Template © CFFT TDN Effective: 12 SEP 2007
